# The complete genome sequence of “*Candidatus* Liberibacter asiaticus” strain 9PA and the characterization of field strains in the Brazilian citriculture

**DOI:** 10.1128/msphere.00376-24

**Published:** 2024-11-11

**Authors:** Michele F. S. Dutra, Priscila A. Silva, Jianchi Chen, Nelson A. Wulff

**Affiliations:** 1 Universidade Estadual Paulista (UNESP), Instituto de Química, Araraquara, Sao Paulo, Brazil; 2 Departamento de Pesquisa e Desenvolvimento, Fundo de Defesa da Citricultura–Fundecitrus, Araraquara, Sao Paulo, Brazil; 3 Universidade Estadual de Campinas, Genomics for Climate Change Research Center, Cidade Universitária Zeferino Vaz, Campinas, Sao Paulo, Brazil; 4 United States Department of Agriculture–Agricultural Research Service, San Joaquín Valley Agricultural Sciences Center, Parlier, California, USA; E O Lawrence Berkeley National Laboratory, Berkeley, California, USA

**Keywords:** genome, “*Candidatus *Liberibacter asiaticus”, prophage, huanglongbing, plant pathogens

## Abstract

“*Candidatus* Liberibacter asiaticus” (CLas) is associated with citrus huanglongbing, a severe disease with global importance that affects citrus production in Brazil. This study reports the first complete genome of a Brazilian strain of CLas. The genomic structure comparison of strain 9PA with those of 13 complete CLas genomes revealed 9,091 mismatches and 992 gaps/insertions, highlighting eight locally colinear blocks, among which six are in the prophage region. Phylogenetic analysis categorized 13 CLas genomes into two clusters with 9PA clustered with strains from China and the United States. Whole-genomic comparison identified diverse hypervariable genomic regions (HGRs). Three HGRs in the chromosomal region and three in the prophage region were selected and investigated by polymerase chain reaction. HGRs assessed from 68 samples, from medium- to high-huanglongbing incidence areas in Sao Paulo state, were grouped into haplotypes A to P. Haplotype A, which includes strain 9PA, is the second most prevalent, representing 19.1% of the samples. Haplotype B, the most common, accounts for 42.6%. Together with haplotype C, these make up 72% of the evaluated samples. The 9PA strain has prophage P-9PA-1, both integrated and circularized, and P-9PA-3, only found in a circularized form. Prophages show high identity with SC1 (83%) and P-JXGC-3 (98%). Co-occurrence of both type 1 and 3 prophages was observed in field samples. The approach employed provides insights into the Brazilian CLas population, providing markers for population studies and highlighting the prevalence of type 1 and 3 prophages in the population.

**IMPORTANCE:**

CLas is a destructive pathogen responsible for causing the severe citrus disease known as huanglongbing. Our study presents the first fully sequenced Brazilian strain of CLas, designated as 9PA, and includes an analysis of two prophages occurring in this strain. The main objective of our research was to compare the genome features of this Brazilian strain with other fully sequenced genomes and to identify its hypervariable genetic regions. These regions were subsequently used to assess genomic variability within both the chromosomal and prophage regions in Brazilian isolates of CLas. Our findings offer valuable insights into the diversified adaptation of CLas.

## INTRODUCTION

Huanglongbing (HLB) is a globally emergent and highly destructive citrus disease (1, 2). Characterized by its devastating effect on citrus crops, HLB has been a long-standing concern for the citrus industry since its discovery in China at the end of the 19th century (3). The disease is attributed to three unculturable α-proteobacteria species within the genus *Candidatus* Liberibacter: “*Candidatus* Liberibacter asiaticus” (CLas), “*Candidatus* Liberibacter americanus” (CLam), and “*Ca*. L. africanus” (2, 4, 5). Among these, CLas has garnered particular attention due to its global prevalence and the extensive damage it inflicts on citrus orchards. In Brazil, CLas remains the primary bacterium responsible for the disease, infecting approximately 99.9% of HLB-infected trees, while CLam is no longer found in field (1).

The inability to axenically cultivate CLas has posed unique challenges, necessitating the use of polymerase chain reaction (PCR)/next-generation sequencing-based techniques to investigate its genetic diversity. A milestone in HLB research was the publication of the first CLas genome (6), enabling comprehensive exploration of the CLas genomic structure and genome-wide analyses, leading to the identification of prophage elements (7). Additional metagenome efforts have offered insights to unravel its genetic diversity using molecular markers (8–15) and key genes involved in host interactions (16–24).

The CLas genomes consists of a highly conserved chromosomal backbone and a variable prophage region. Prior studies investigating CLas genetic variations have primarily focused on restriction fragment length polymorphism (RFLP), single-nucleotide polymorphisms (SNPs), and indels in specific regions, such as 16S/23S ribosomal DNA, deoxyribonucleotide reductase gene, the *omp* gene region, the β-operon gene loci, or hypervariable prophage regions (11, 12, 14, 25–37). Another source of variability is the short tandem repeats (STRs), widely studied in CLas as well (8, 11, 38–44). While minimal genetic variations were observed within the analyzed non-prophage regions, the prophages displayed significant hypervariable regions that played a crucial role in population diversity studies (12, 14, 36, 37, 45).

The availability of 46 genome sequences for CLas, including 13 fully complete ones, has substantially advanced our ability to identify these hypervariable genomic regions (HGRs) and conduct comprehensive studies on the genetic diversity within CLas populations (37, 46, 47). Prophages are unique sources of variability. Exploring CLas genomes and their prophages is of crucial significance in unraveling insights into the HLB disease. Lysozymes, which are important for phage liberation from hosts; repressors, whose product binds to operators in the prophage to prevent the initiation of the lytic cascade of gene expression; and peroxidases, which aid bacteria in evading reactive oxygen species produced by the infected plants or insects, are prophage-encoded products with roles in bacterial adaptation and survival (19, 20, 48).

In this study, we report the complete genome of representative CLas strain 9PA, the first to be completely sequenced from Brazil, extending from the previous draft genome sequence (49). This sequence was compared to other complete genomes to investigate genomic structure, such as locally colinear blocks (LCBs) and potential genetic variations, such as HGRs. A set of samples from HLB regions from São Paulo State (SPS) was subjected to PCR using primer sets targeting six HGRs, including the presence of prophages from type 1 and type 3 in such strains. This study aims to provide a deeper understanding of the dynamics of the CLas population within the context of Brazil.

## RESULTS

### The complete 9PA genome sequence

The 9PA draft genome consisting of 157 contigs and the integration of unpublished data from the same metagenome sequencing run, consisting of 135 additional contigs, were all aligned in relation to the psy62 strain. Furthermore, PCR for the gap-closure strategy yielded a total of 124 PCR products, and, following Sanger dideoxy terminal dye sequencing, a collection of 209 trimmed reads ranging from 59 bp to 1,097 bp was generated. These reads closed all gaps in the draft 9PA genome. Special attention was devoted to the amplification of a ribosomal operon, *rrnB* from draft 9PA assembly, since SNPs were found in this region in the draft assembly. The 7,962-bp sequence, covering the *rrnB* operon, displayed uniformity with other CLas ribosomal operons, suggesting a lack of intra-operon variability. Consequently, it was employed to confirm the 5S, 16S, and 23S regions of both *rrnA* and *rrnC*, as they share identical sequences, and integrated into the 9PA assembly. Collectively, Sanger reads accounted for a total of 183,294 bp, constituting 14.9% of the 9PA genome.

**Fig 1:**
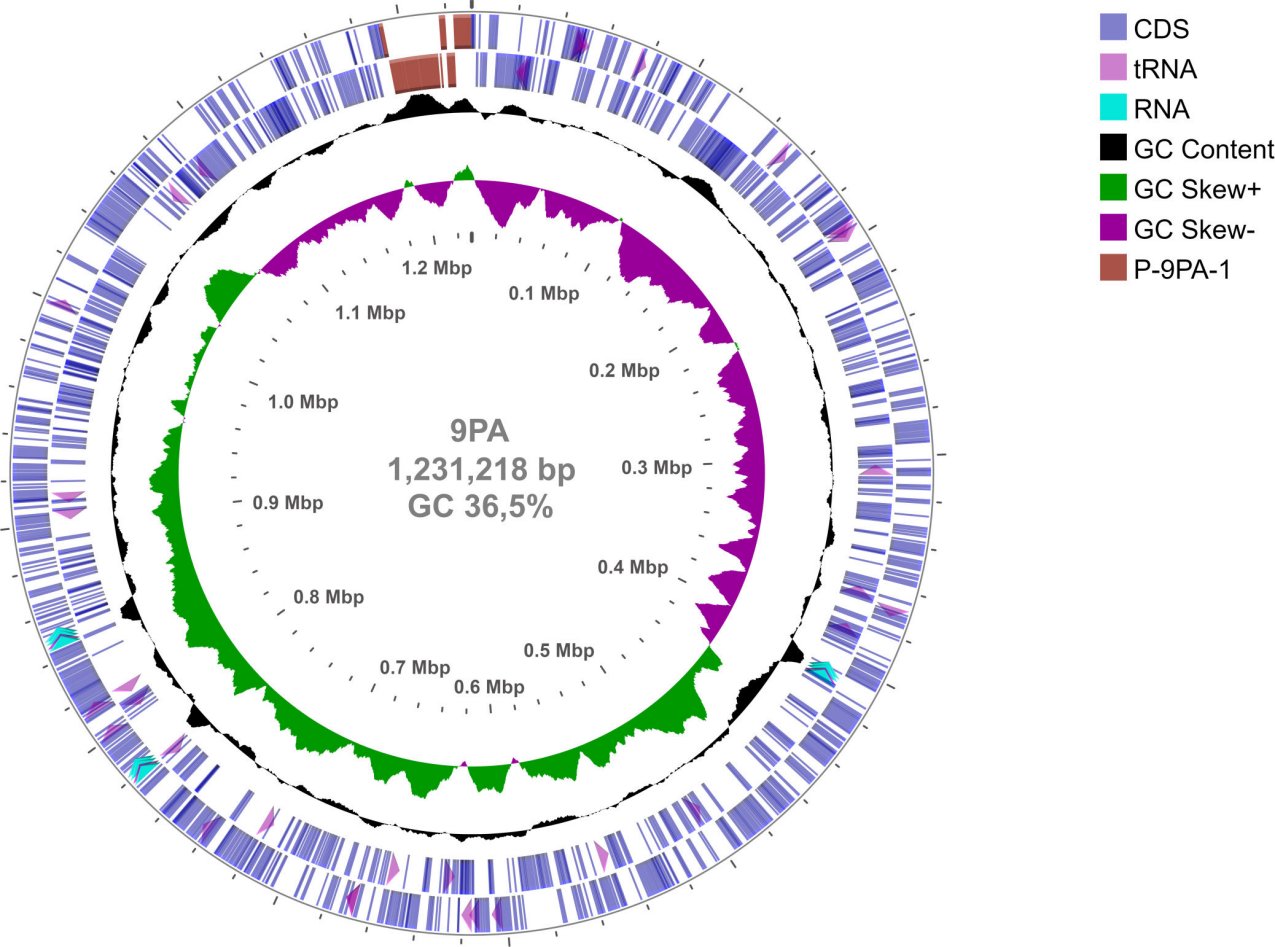
Schematic representation of 9PA strain “*Candidatus* Liberibacter asiaticus” genome. Circular representation of the 1,231,218-bp genome. The tracks from circles represent forward and reverse coding sequences (CDS) (blue), P-9PA-1 prophage region (dark red), rRNA operon (light blue), tRNA (rose), % G+C content (black), and GC skew [G − C/(G + C)] (green and purple).

Ultimately, the strategy culminated in the successful assembly of a circular chromosome (accession number CP145497.1) measuring 1,231,218 bp with a GC content of 36.5% (Fig. 1). The Prokaryotic Genome Annotation Pipeline (PGAP) annotation unveiled 1,111 coding sequences, of which 1,025 are protein coding, encompassing 42 from the prophage region. Additionally, 53 RNAs were identified, including 44 tRNA molecules and three complete ribosomal operons.

### Comparison of CLas strain genomes

**Fig 2:**
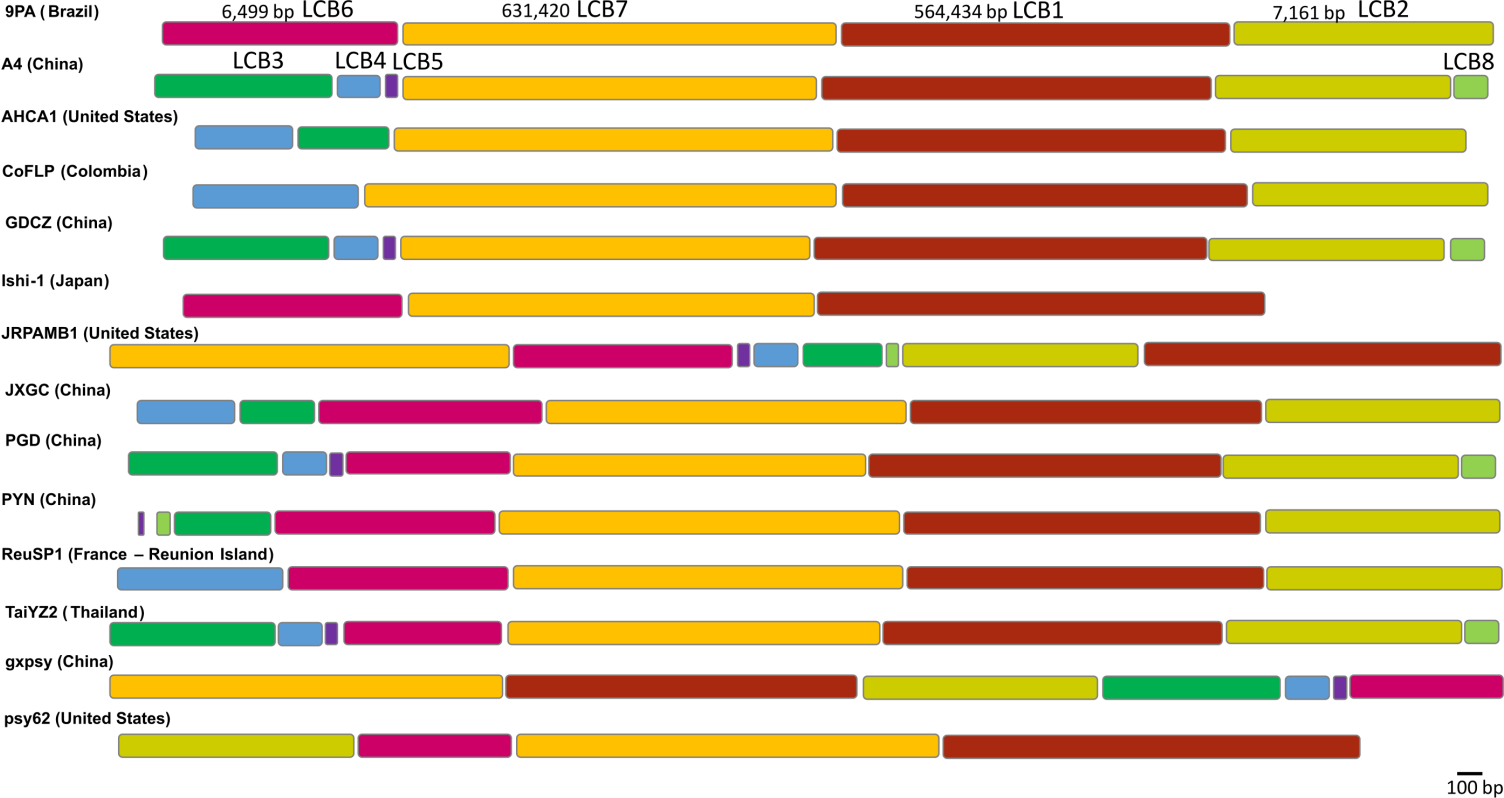
Locally colinear blocks (LCBs) identified among 14 *Candidatus* Liberibacter asiaticus genomes. Each contiguous and color-coded region represents an LCB, denoting a segment without rearrangements in the homologous backbone sequence. To enhance clarity, the LCBs are not presented in the same proportion ratio. The two major LCBs are delineated by dark red (LCB1) and yellow (LCB7) rectangles in a proportion of 1:400. LCB2 (mustard) and LCB6 (rose) are displayed in a proportion of 1:7. The remaining LCBs, LCB3 (green), LCB4 (blue), LCB5 (purple), and LCB8 (light green), are shown in a proportion of 1:1.

The CLas 9PA genome strain sequence was compared to 13 complete CLas available genomes revealing eight LCBs (Fig. 2). LCB1 (564,434 bp, spanning from base 637,063 to 1,223,426 in reference to the 9PA genome) exhibited 92.4% pairwise identity, with only 73.5% of identical sites due to variations caused by the prophage region. By contrast, LCB7 (631,420 bp, from base 5,643 to 637,062) demonstrated a high similarity, with 99.5% identical sites and 97.7% pairwise identity. Two of these LCBs together constitute a substantial 97.6% of the CLas-compared genomes. The remaining LCBs are located within the prophage region and exhibited distinct identities and distribution across genomes: LCB2 (7,161 bp: 96.7% pairwise identity and 90.2% identical sites; absent in Ishi-1), LCB3 (702 bp: 65.8% pairwise identity and 37.9% identical sites; absent in CoFLP, 9PA, Ishi-1, psy62, and ReuSP1), LCB4 (172 bp: 35.9% pairwise identity and 19.8% identical sites; absent in psy62, 9PA, PYN, and Ishi-1), LCB5 (49 bp: 96.4% pairwise identity and 89.8% identical sites; absent in 9PA, Ishi-1, AHCA1, GDCZ, psy62, CoFLP, and JXGC), LCB6 (6,499 bp: 98.1% pairwise identity and 91.6% identical sites), and LCB8 (135 bp: 89.9% pairwise identity and 86.7% identical sites). Notably, LCB3, LCB4, LCB5, and LCB8 are repetitive regions comprising fragments of the DNA helicase gene, which marks the site of prophage insertion into the genomes. Mismatches between genomes were 9,091, and gaps/insertions were 992, including the prophage region.

**Fig 3:**
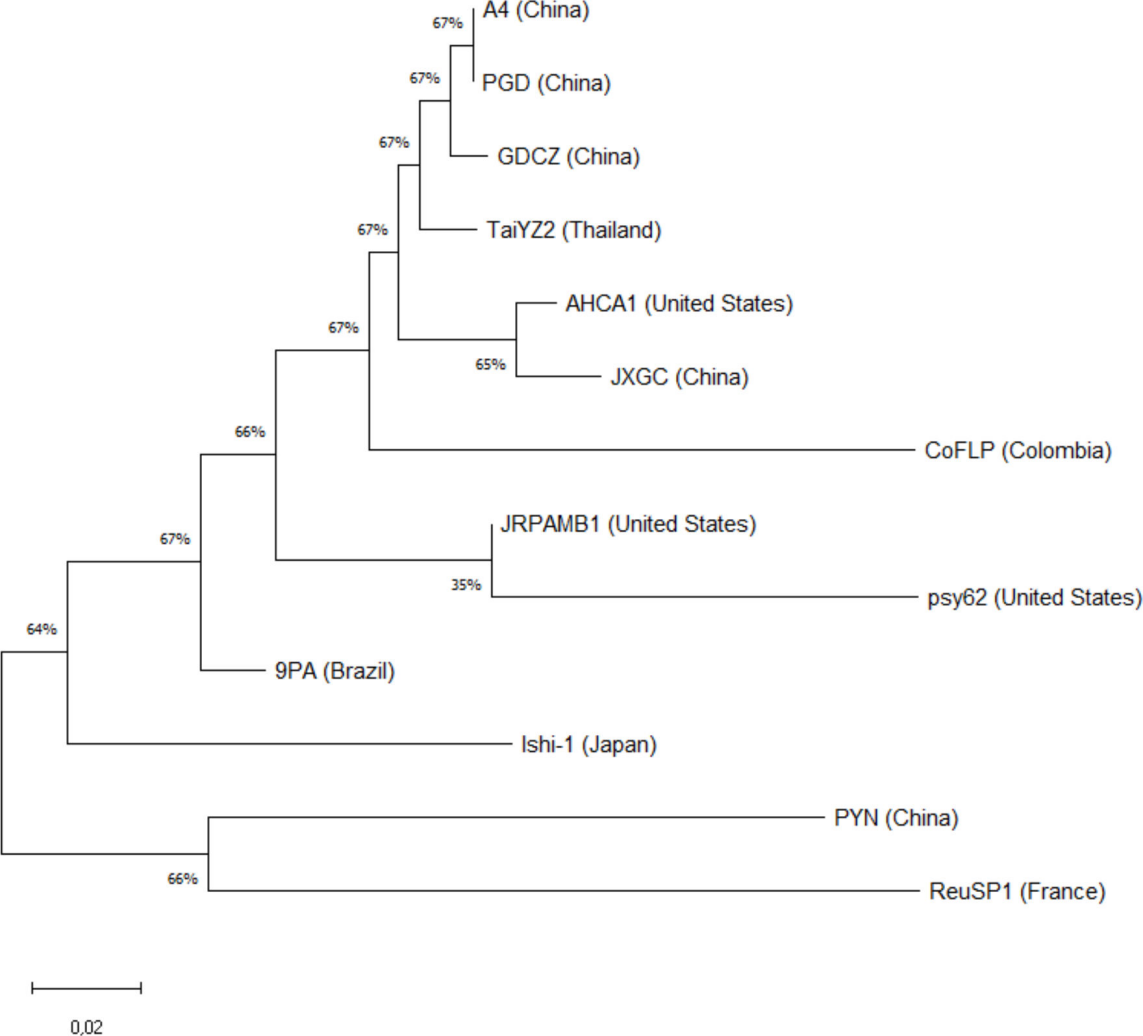
Phylogenetic tree of 13 *Candidatus* Liberibacter asiaticus genome strains. The phylogenetic tree was constructed using neighbor joining. The optimal tree is shown with the gxpsy strain not aligned to the group. The tree is drawn to scale, with branch lengths in the same units as those of the evolutionary distances used to infer the phylogenetic tree. The evolutionary distances were computed using the *p*-distance method and are in the units of the number of base differences per site. The proportion of sites where at least one unambiguous base is present in at least one sequence for each descendent clade is shown next to each internal node in the tree. Codon positions included were first + second + third + non-coding. All ambiguous positions were removed for each sequence pair (pairwise deletion option).

Unrooted neighbor joining (NJ) was used to uncover the phylogenetic distance and the genetic variability between complete CLas genomes. Through pairwise deletion, ambiguous positions were excluded, culminating in 1,846,468 definitive positions (Fig. 3). The analysis distinctly reveals two divergent groups stemming from a common ancestor. On one side, a cluster is formed by representatives from China (A4, PGD, GDCZ, and JXGC), Thailand (TaiYZ2), the United States (AHCA1, JRPAMB1, and psy62), Colombia (CoFLP), Brazil (9PA), and, more unrelated, Japan (Ishi-1), a strain without prophages of types 1, 2, and 3. Notably, strains A4, PGD, GDCZ, TaiYZ2, AHCA1, and JXGC constitute a closely related subcluster within this grouping. On the other side, the most divergent cluster is formed by a representative from China (PYN) and one from Réunion Island, France (ReuSP1). This grouping underscores the divergence observed among strains mostly from Asia and the broader group. Additionally, the representative from Brazil (9PA) appears more closely related to the U.S. strains JRPAMB1 and psy62. Due to the organization of LCBs in the CLas gxpsy strain, this genome was not incorporated into the genetic variability analysis and consequently in the unrooted tree.

Among the 14 compared genomes, 252 differences greater than 20 bp were identified. Subsequently, analysis revealed six HGRs, defined by their presence in three or more genomes (Table 1). Notably, three HGRs are situated within the chromosomal region, while three reside within the prophage region. These prophage-situated HGRs hold promise as valuable markers for distinguishing between different types of prophages.

**TABLE 1:** HGR^*a*^ characteristics in “*Candidatus* Liberibacter asiaticus” genomes

HGR	Location in 9PA genome	Length	Gene product (gene ID)
2	976,152–978,290	2,139 bp	Phage repressor protein/BRO family protein (HF993_04350) and hypothetical proteins (HF993_04355 and HF993_04360)
4	1,216,261–1,216,553	293 bp (type 1 prophage) 601 bp (type 2 prophage) 624 bp (type 3 prophage)	Transcriptional regulator/phage-associated repressor protein C2 (HF993_05470)
6	1,204,251–1,204,271	21 bp (type 1 prophage)	Prophage cell envelope integrity protein TolA/colicin Ia (HF993_05415)
7	1,191,721–1,193,120	1,400 bp (type 1 prophage) 2,290/2,593 (type 2 prophage)	Guanylate kinase (P9PA1_gp41) and hypothetical proteins (P9PA1_gp42, P9PA1_gp01 and P9PA1_gp02)
12	346,667–346,701	Variable	Tandem repeat (AGACACA) in the transcriptional regulator/phage repressor protein (HF993_01565)
M	1,078,210–1,079,679 and 1,034,133–1,034,515	Variable	*Microviridae* CLasMV1(HF993_05375, HF993_05380, HF993_05385, and HF993_04595)

^*a*^ HGR, hypervariable genomic region.

**Fig 4:**
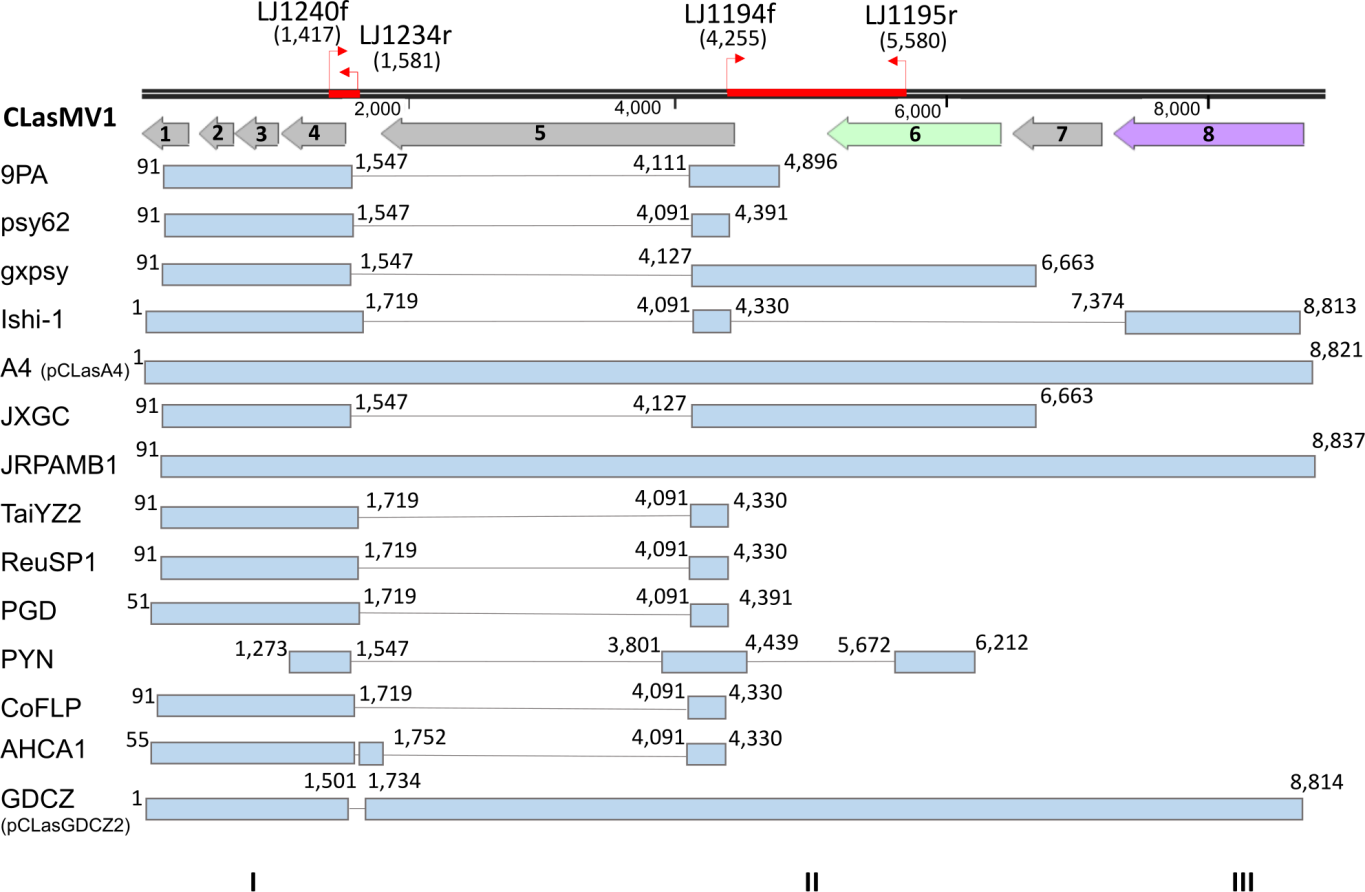
Presence of CLasMV1 within genomes of “*Candidatus* Liberibacter asiaticus,” identified as HGR_M. Light blue boxes represent regions of sequence similarity between CLasMV1 and the CLas genomes, indicating significant hits. The numbers indicate specific start and end positions where hits were detected. Gray boxes correspond to hypothetical ORFs 1–5 and 7 of CLasMV1, and light green and light purple boxes (6 and 8) represent the major capsid protein and the replication initiation protein, respectively. Red arrows mark the regions of genomes where primers LJ1194f/ LJ1195r and LJ1240f/ LJ1234r anneal. The positions of the primer annealing sites are enclosed in parentheses, indicating their initial and final positions in the CLasMV1 genome, and the three mean regions of BLASTn hits are represented by I, II, and III.

HGR_2 spans a chromosomal located sequence of 2,139 bp, coding for two hypothetical proteins and a partial C-terminal fragment of a BRO family protein (HF993_04350), also annotated as a phage repressor, which is absent in both the JRPAMB1 and psy62 genomes. It is worth mentioning that HGR_2 was found to be absent in the 9PA draft metagenome in the assembly previously obtained. Through subsequent PCR validation, however, the missing sequence was successfully incorporated into the 9PA genome sequence. HGR_4 represents a segment encompassing a variable prophage region that is part of a putative C2 transcriptional regulator/phage-associated repressor protein (HF993_05470). Despite being located within the early-gene region, this sequence demonstrates a divergent distribution pattern across the different types of prophages: 293 bp for the type 1 prophage, 601 bp for type 2, and 624 bp for type 3. HGR_6 comprises a 21-bp sequence within an open reading frame (ORF) encoding a prophage cell envelope integrity protein, alternatively annotated as a putative colicin Ia (HF993_05415). Notably, by sequence analysis, this ORF was detected only in strains carrying a type 1 prophage, such as gxpsy, psy62, ReuSP1, and PYN. Considering, however, that primer 6_F was specifically designed in the 21-bp sequence showing divergences in occurrence in the evaluated CLas strains, it is possible that strains harboring the type 1 prophage might not exhibit the amplification pattern as a result. HGR_7 displays variability within the prophage regions across strains. The sequence is positioned at the juncture of the early-gene and late-gene regions and shows two patterns of amplification: 1,400 bp for the type 1 prophage and 2,290/2,593 bp for the type 2 prophage. In 9PA, the type 1 prophage sequence comprises a mosaic region with a guanylate kinase (P9PA1_gp41) and three hypothetical proteins (P9PA1_gp42, P9PA1_gp01, and P9PA1_gp02). Even among strains hosting the same types of prophages (type 1 or 2), distinct amplification patterns can be obtained. HGR_12 is a variable tandem-repeat chromosomal region (AGACACA) that has been previously highlighted in genetic studies involving CLas strains (38, 41, 44). This region resides within an ORF responsible for encoding a transcriptional regulator/phage repressor protein (HF993_01565). In addition, HGR_M stands out as it is linked to a recently identified member of the CLas *Microviridae* family (46, 50). The phage, named CLasMV1 (GenBank accession number CP045566.1), harbors eight ORFs and was identified as a variable region within CLas strains, with only two (JRPAMBI and A4) harboring the full sequence (Fig. 4).

### Occurrence of six HGRs in CLas strains from a field in central and south/southern São Paulo state, Brazil

**Fig 5:**
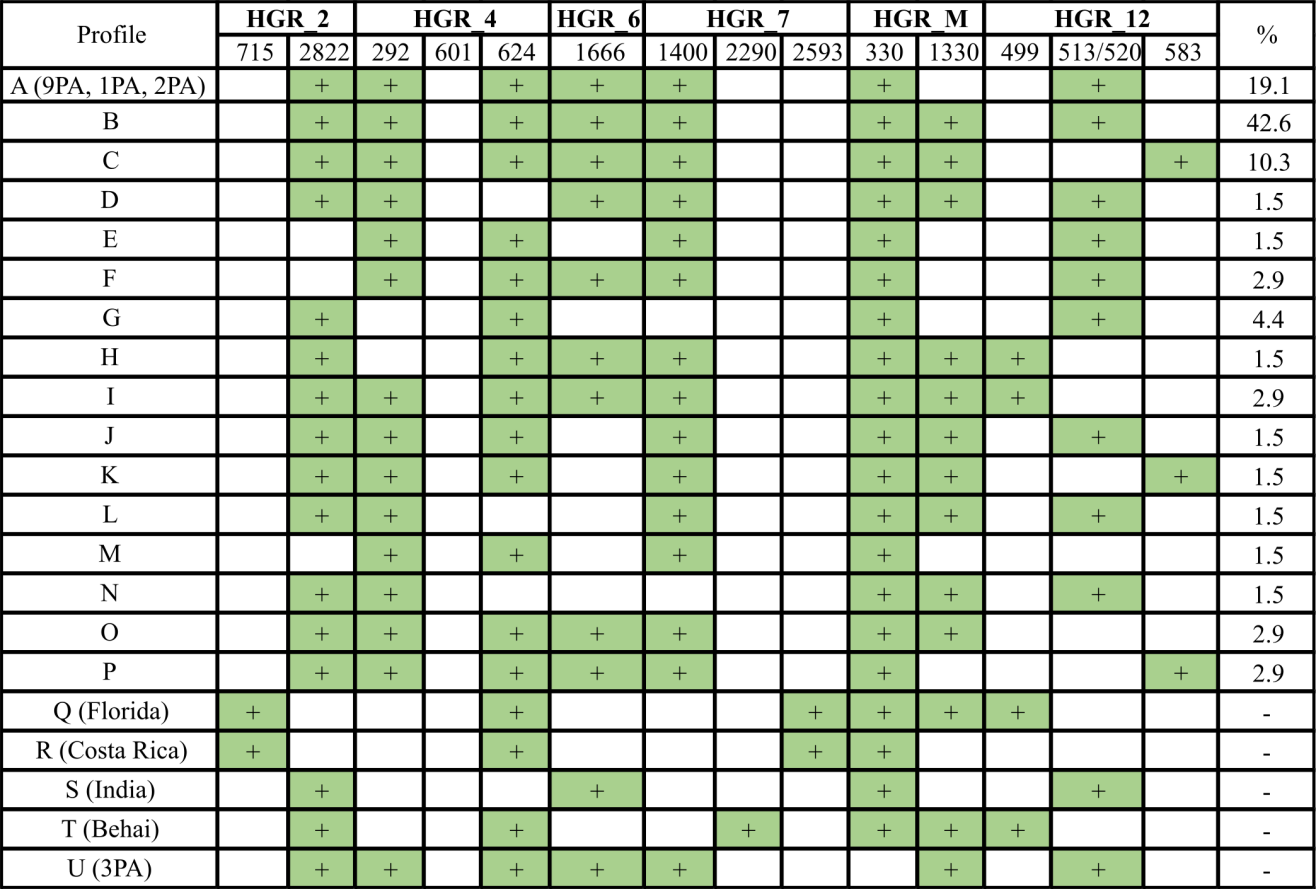
Classification of 68 field strains and control samples of CLas categorized into distinct profiles based on the patterns of PCR amplifications observed for the six hypervariable genomic regions (HGRs) evaluated. The numbers below HGRs are related to the expected size of PCR products. Green is presence, while white is absence.

In this study, a collection of 68 symptomatic samples from 12 localities within five areas of medium and high incidences of HLB in SPS, had low cycle threshold values and PCR amplicons consistent with the presence of CLas infection (Table S1; Fig. S1). In our analysis of six HGRs, these CLas field and also greenhouse-kept strains were categorized into 21 distinct profiles, from A to U, according to the occurrence and size of the amplification products representing each HGR (Fig. 5).

The 68 field strains represent 16 profiles, from A to P, with 19.1% displaying profile A, which was found to be identical to the 9PA strain profile. Another 42.6% of the strains presented profile B, with a single difference in relation to profile A, in the HGR_M, while 10.3% had profile C (Fig. 5). The remaining 27.9% of the strains exhibited a diverse range of 13 different profiles, labeled from D to P. Within this group, profiles F, I, O, and P each had two representative strains, while profile G had three representative strains. Finally, profiles D, E, H, J, K, L, M, and N were each represented by one sample. Among 68 CLas strains, 94.1% showed amplification for HGR_2, while 5.9% did not show amplification for this region (Fig. S2; Table S2). In case of HGR_4, 89.7% of strains presented concomitantly fragments of 293 and 624 bp, meaning both type 1 and type 3 prophages were present. In the remaining strains, 5.9% and 4.4%, respectively, presented amplifications of only 624 bp (type 3) or 293 bp (type 1), thus indicating that while ~90% of strains had sequences of two prophages, the remaining ~10% had sequence of only one. Regarding HGR_6, 86.8% of the strains exhibited amplification, while 13.2% lacked the 21-bp variation, where no 1,666-bp amplification occurs. Similarly, for HGR_7, 94.1% of the strains displayed amplification of 1,400 bp, characteristic of type 1 prophages. No field strain presented PCR amplification of HGR_7 pattern for type 2 prophage (2,290 or 2,593 bp), except for the control strains from Behai in China, Costa Rica, and Florida in the United States. Additionally, two strains did not exhibit any amplification in HGR_7, as observed in the control strain from Poona in India.

The HGRs 4, 6, and 7 collectively provide a comprehensive framework for delineating prophage occurrences in CLas strains. The identification of type 1 prophage is marked by the amplification of a 293-bp fragment. The ambiguity between 624 bp (type 3) and 601 bp (type 2) can be resolved through targeted amplification of 1,666 and 1,400 bp for HGR_6 and HGR_7, respectively, a possibility only in the presence of type 1 prophage. In instances where type 2 prophage is present, a distinctive fragment of 2,290 or 2,593 bp is amplified, as observed in strains from Behai, Costa Rica, and Florida. Additionally, we observed a predominant 1,400-bp amplification pattern characteristic of type 1 prophages (HGR_7), even in a sample that amplified only a 624-bp fragment in HGR_4 (sample 30), meaning that more than one strain may be present in a single tree.

Specifically, HGR_12 presented three distinct possibilities based on genome comparison and amplicon size: (i) 499 bp, (ii) 513–520 bp, and (iii) 583 bp. Approximately 76.5% of the strains had the intermediate amplicon (513–520 bp), while 14.7% exhibited amplifications for the 583-bp PCR product, and 4.4% showed amplifications for the smaller amplicon (with 499 bp). The remaining 4.5% of the strains had no amplification of this marker (Fig. S2; Table S2).

In the context of the screening analysis for HGR_M, two distinct profiles were identified. The presence of region I, spanning from bases 1 to 1,719 (or 91–1,547 concerning 9PA), consistently resulted in the amplification of a 333-bp fragment, observed in 100% of the analyzed strains. Intriguingly, when both region I and the complete region II (encompassing positions from 4,091 to 6,663) were present, amplification produced fragments of both 333 and 1,333 bp. Notably, in our sample set, 67.6% exhibited the concurrent presence of regions I and II, while none had the complete region II alone. The full sequence of CLasMV1 can be deduced by analyzing the PCR data obtained using the primer set LJ1240f/LJ1234r. This set amplifies a unique 165-bp fragment specifically when the entire CLasMV1 sequence is present within the CLas genome (data not shown).

Regarding the control strains kept *in planta* in the greenhouse or received as DNA samples, they exhibited distinct profiles. More specifically, 9A, 1PA, and 2PA were grouped into profile A, while Florida (USA) was identified as profile Q, Costa Rica as profile R, India as profile S, Behai (China) as profile T, and 3PA as profile U. Despite being classified as distinct profiles, Florida and Costa Rica exhibited a strikingly similar pattern of amplification, with just two differences, specifically at HGR_M and HGR_12. The Costa Rica sample only showed amplification of 330 bp for HGR_M and no amplification for HGR_12 (Table S2).

### Prophage characterization

Previously described as type 1 and 3 prophages (49), contigs 156 and 157 of 9PA draft genome were designated, respectively, as P-9PA-1 (accession number: PQ160487.1) and P-9PA-3 (accession number PQ160474.1) according to established nomenclature (51). P-9PA-1 genome is 39,767 bp and the GC content is 41.46%, while P-9PA-3 is 31,802 bp and the GC content is 39.93% (Fig. S3).

**Fig 6:**
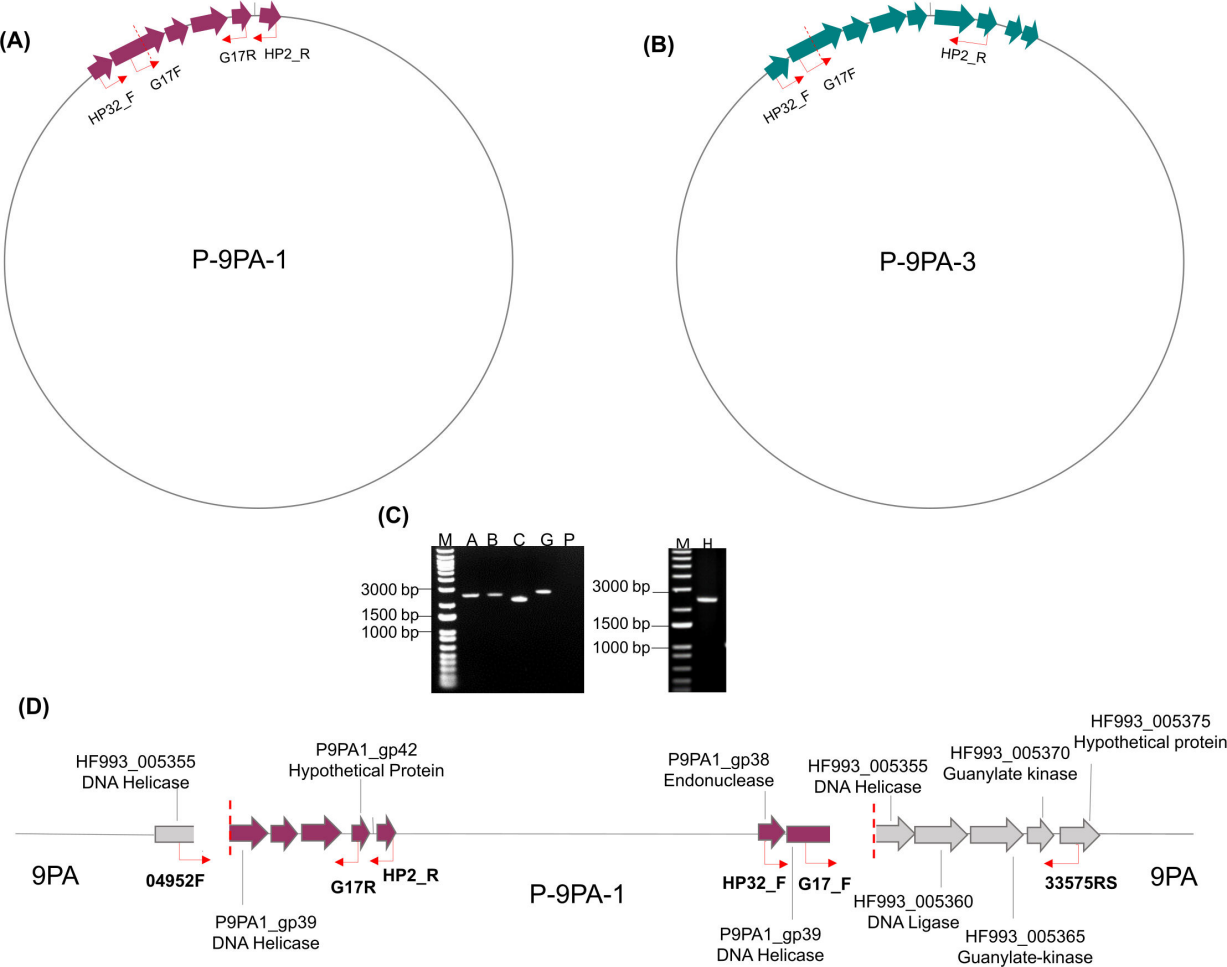
Prophages P-9PA-1 and P-9PA-3 organization into strain 9PA genome. (**A **and **B**) Circular forms of prophages. (**C**) Amplification fragments obtained while using the primer sets A (G17F/G17R—2,608 bp), B (04952F/G17R—2,616 bp), C (G17F/33575RS—2,210 bp), G (G17F/HP2_R—2,832 or 4,125 bp), P (04952F/33575RS—2,218 bp), and H (HP32_F/33575RS—2,446 bp). Lane M is 1 kpb plus marker from Invitrogen. (**D**) Linear form (chromosomal integration) of P-9PA-1. The region of integration is flanked by two red dashed lines. The dark red and the gray arrow boxes represent the prophage genes and the chromosomal genes, respectively. The red arrows indicate the primer annealing region. Six primers were combined for determining the presence of circular and linear forms of prophages.

PCR amplifications with specific primers based on regions of prophage integration in CLas chromosome indicated that P-9PA-1 occurs both as circular (primer sets A and G) and integrated prophages (primer sets B and C), while P-9PA-3 occurs only as circular (Fig. 6).

**Fig 7:**
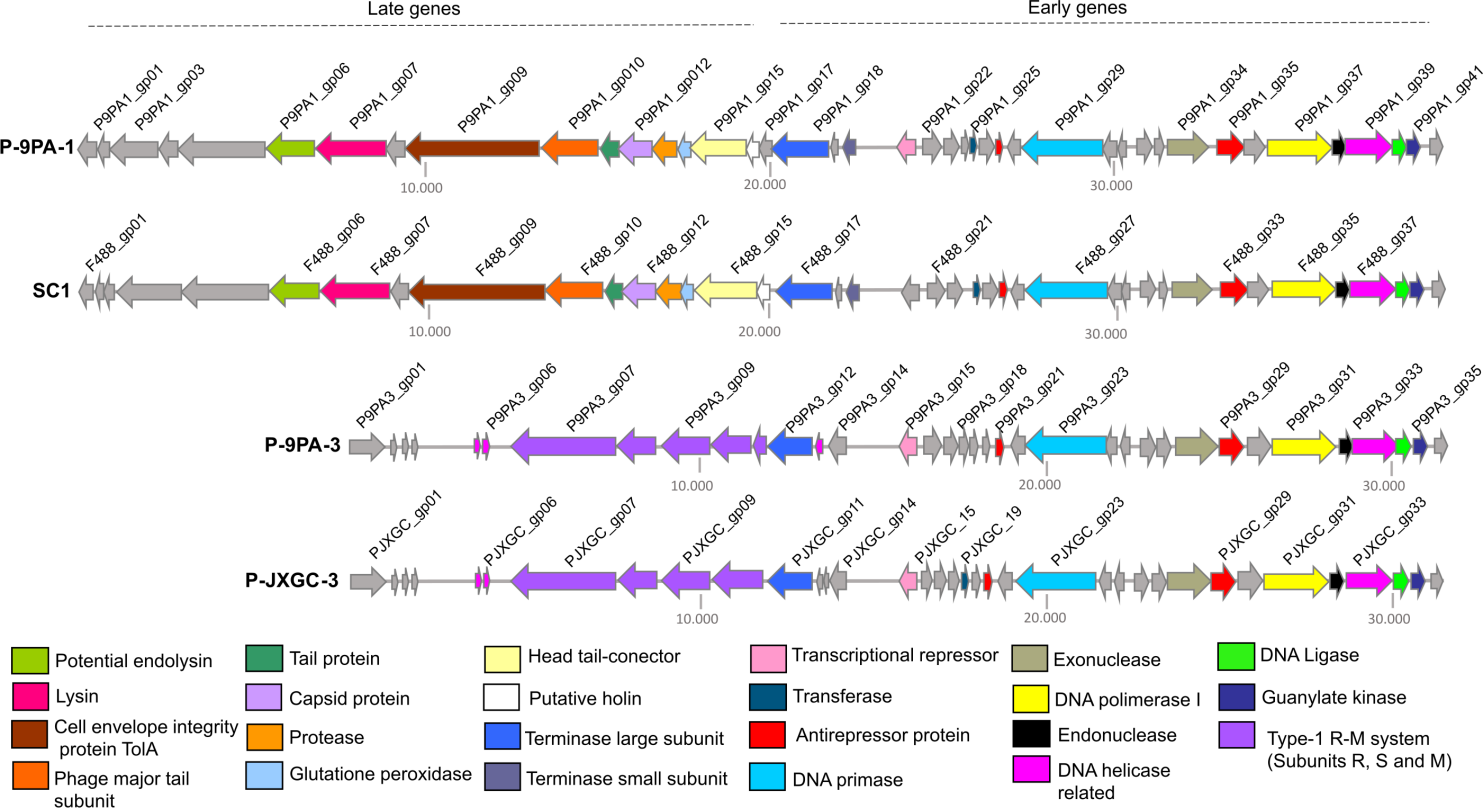
Genomic alignment map depicting the similarities between prophages of “*Candidatus* Liberibacter asiaticus“ strain 9PA to SC1 from UF506 and P-JXJC-3 from JXGC strains. Annotated genes sharing identical functions are represented by arrow boxes of the same color.

The prophage P-9PA-1, comprising 42 ORFs, displayed sequence identities of 83%, 65%, and 67% when aligned with SC1, SC2, and P-JXGC-3, respectively. In comparison with SC1, 841 differences were noted, including 815 mismatches and 26 gaps/insertions. Four ORFs, P9PA1_gp03, P9PA1_gp04, P9PA1_gp12, and P9PA1_gp17, showed significant nucleotide-level differences when compared to SC1 and matched rearrangements and identities with other ORFs from CLas type 1 prophage. Despite the rearrangement of both prophages in the late-gene region, this region was nearly identical with 91% sequence identity (Fig. 7; Table S3). In the early-genes region, common among most CLas prophages, P-9PA-1 and SC1 exhibited 92% of identity, differing in only three ORFs: P9PA1_gp20, P9PA1_gp21, and P9PA1_gp41. These ORFs matched similarities with P-JXGC-3 and other CLas type 1 prophages, including types C, D, H, and G prophage regions of strain FL-Periwinkle (37).

Moving on to prophage P-9PA-3, which comprises 36 ORFs, we found that alignment with P-JXGC-3 revealed 112 mismatches and nine gaps, primarily in non-coding regions and in early genes (P9PA3_gp15 and P9PA3_gp16). These ORFs showed identities to type C, D, and H prophage regions of FL-Periwinkle and to type 1 and 2 prophages. Despite these variations, the prophages shared an impressive 98% identity in the global alignment, with the remaining ORFs being identical (Fig. 7; Table S3). Similarities to SC1 and SC2 prophages were 65%, mostly due to the early-gene region.

Both P-9PA-1 and P-9PA-3 had three spacers in the clustered regularly interspaced short palindromic repeats/*cas* system (CRISPR/Cas) within the hypothetical proteins P9PA1_gp30 and P9PA3_gp24 (15, 24). Additionally, a restriction–modification (R-M) system with subunits R, S, and M was found only in P-9PA-3 (P9PA3_gp07–P9PA3_gp11; Fig. 7).

## DISCUSSION

Draft genomes are highly useful for initial inquiries and exploratory investigations, but for comprehensive genomic structure studies, complete genomes provide further biological information (52). In our research, we closed gaps of the 9PA draft genome (49) using PCR amplifications and Sanger sequencing, resulting in a circular 1,231,218-bp genome.

In a genome-wide comparative analysis, we scrutinized the genetic landscapes of the 9PA CLas genome strain in comparison with 13 complete CLas genomes, encompassing strains from China, United States, Colombia, La Réunion in France, Japan, and Thailand. Our analysis revealed the presence of eight LCBs, regions of genomes that share conserved synteny. Since CLas is an obligate internal pathogen, the conservation of these genomic structures suggests a vital role in the pathogen’s biology and interaction with its hosts. Besides two major LCBs in the chromosomal region, six LCBs were located within the prophage, indicating susceptibility to genetic rearrangements under evolutionary pressures from dynamic environments (53). Other than the prophage sequences, the genomes maintained a high identity at the chromosomal level, meaning that divergence in evolutionary terms is recent.

Beyond these genomic comparisons, our study allowed the identification of six hypervariable genetic regions. In the prophage region, screenings of HGR_4, HGR_6, and HGR_7 revealed a significant co-occurrence of both type 1 and 3 prophages in a substantial majority of our examined samples, instead of the unique presence of either type 1 or 3. This observation aligns with the conclusions drawn from CLas strains from China, where the combination of type 1 and 3 prophages was the second most frequent of a total of eight combinations, with type 1 or 2 alone being the most common (24). The study also correlated the co-occurrence of type 1 and 3 prophages with the presence of an R-M system (present in P-9PA-3) and a CRISPR/*cas* system (present in both P-9PA1 and P-9PA-3). It has been suggested that CLas uses type 3 to defend against type 1 prophage invasion, as both systems are employed by bacterial hosts for protection against phage invasions (24). In this case, the presence of type 1 prophage exerts a positive pressure to select CLas strains that harbor type 3 prophages (24). Previous studies of Brazilian CLas strains assessed the presence of only type 1 and 2 prophages yet, consistent with the current work, found that all the strains evaluated were found to contain prophages (44). Additionally, recent research has revealed additional, previously uncharacterized prophages and prophage-like sequences (17, 54). This suggests that other uncharacterized types should also be considered, even in the absence of known prophages. Prophages are a primary source of genetic variation among CLas strains (15, 47). Although they can play a role in enhancing the virulence of CLas, they are not critical for its pathogenicity or survival (17). Importantly, the presence and diversity of prophages reveal significant regional differences in CLas strain composition, as demonstrated by research from China, India, the United States, and Pakistan (28, 39, 47, 54–56) and have been used to trace origins or compare evolutionary dynamics (36, 56).

In the chromosomal region, we detected the presence of HGR_2, a feature missing in the draft genome of strain 9PA (49). This discrepancy is most likely attributed to inaccuracies within the next-generation sequencing data or assembly tools, since the same plant source was used and that 94.1% of the SPS field samples contained this HGR, highlighting the importance of the complete genome sequence. On the other hand, this HGR was absent from samples from Florida (USA) and Costa Rica. For HGR_12, the region in 9PA shows similarities with the pattern found for strains JRPAMB1, psy62, and PYN and suggests potential regional genetic signatures within CLas populations (38), underscoring genetic heterogenicity. Although the complete CLas *Microviridae* (50) was not identified in the SPS strains (HGR_M), a consistent pattern emerged, indicating the prevalent presence of both regions I and II. Notably, region I demonstrated exceptional significance by being consistently identified in all field screened strains. The diversity observed in these HGRs may have significant implications for CLas genome plasticity. The reduced susceptibility to HLB disease in grapefruit was linked to the absence of an ~8-kb segment from CLasMV1 in those plants (46). Further research is warranted to investigate the functional roles of the other hypervariable regions and their potential effect on bacterial–host dynamics.

The 9PA strain is classified as haplotype A, the second most prevalent under field conditions of SPS, where three haplotypes represent three quarters of the population but with 13 distinct profiles in the divergent quarter. Variations in profiles were also observed in the genomic region with a hypersequence variation in strains from China and Florida, USA (57). The presence of such genetic variations among CLas São Paulo strains may seem surprising, given previous studies that suggested genetic homogeneity within CLas populations in Brazil, particularly using STRs (8, 44). One possible explanation for this variation is the difference in resolution and sensitivity between the methods used in those earlier studies and the whole-genome sequencing and comparative-genomics approach employed in our analysis, which provides a more comprehensive view of the genetic landscape of CLas strains by examining the entire genome, including both coding and non-coding regions and prophage and non-prophage regions. This approach allows us to detect genetic variations that may not be apparent when focusing solely on STRs. A necessary follow-up should be the comparison of distinct ways of performing the molecular characterization of field CLas strains, also considering the possibility of multiple introductions of CLas strains in a single tree (37).

Our analysis of the CLas strain 9PA genome unveiled two distinct prophages: P-9PA-1 and P-9PA-3. Despite being highly similar, a mosaic region was found when compared to their homologus sequence. Like our findings, the mosaic configuration pattern was noted in the new types of prophages from Pakistan CLas strains (54) and in previous studies of CLas populations in Brazil (44). This pattern suggests genetic exchanges and rearrangements, possibly reflecting the dynamic nature of phage genomes (58, 59, 60). Prophage integration in CLas strains was not totally elucidated. Previous studies have reported integration events near a guanylate kinase gene (7), while another study identified a helicase gene as an integration site facilitated by the integrase gene downstream (7, 24). Using primers to assess these previous described loci, we found P-9PA-1 to be both integrated and circular, while we found P-9PA-3 only in the circular form. This reflects the prophage/phage behavior and its potential function within the host, as previously described in CLas strains (7, 24, 54).

Despite the challenges posed by its unculturable nature, advances in whole-metagenome sequencing have allowed us to delve into the genetic intricacies of CLas. Our in-depth analysis of the CLas genome, with a focus on the unique characteristics of the 9PA strain compared to other complete genomes, has provided valuable insights into the diversity of this devastating citrus pathogen responsible for HLB. Additionally, we have uncovered distinct genomic profiles among CLas field strains, with the 9PA strain aligning with one of the representative profiles, highlighting the genetic complexity within the CLas population in Brazil. Importantly, the presence of prophage elements, such as phage-repressor and antirepressor proteins, CRISPR/*Cas* and R-M systems, mosaic-prophage regions, and a new characterized *Microviridae* phage, may play roles in CLas host colonization, survival, and competitivity, consistent with the significance of phage-related elements in CLas biology (37, 50, 55). In summary, our discoveries shed light on the genetic diversity and dynamics of CLas, providing valuable insights to assess the evolution of field strains under agricultural conditions as well as to consider when developing targeted strategies for Liberibacter control. By unraveling the genetic mysteries of this destructive citrus pathogen, we take significant steps toward safeguarding the global citrus industry from the devastating effect of HLB.

## MATERIALS AND METHODS

### 9PA genome gap closure using polymerase chain reaction

The CLas 9PA isolate is maintained in sweet orange (*Citrus sinensis*) since 2010 in greenhouse at the Fundecitrus in Araraquara, São Paulo, and exhibits characteristic symptoms of HLB. This strain was transmitted once using *Diaphorina citri* in 2013, and this original tree is still maintained. Additional propagation was done by graft transmission to healthy nursery trees. Total DNA was extracted from the midrib of its leaves using the cetyltrimethylammonium bromide method (CTAB) (61).

To bridge gaps between contigs in the draft genome of the CLas 9PA strain (49), a combination of PCR amplifications and subsequent Sanger sequencing was employed. Primers (Table S4) were designed between contigs using CodonCodeAligner (version 11.0.1, Dedham, MA), spanning genomic regions from 1 to 6 kbp for the 157 contigs ordered against reference Las genomes (49). In addition, unpublished sequencing data from the draft genome of 9PA (49), consisting of 135 contigs, were included in the assembly. To enhance efficiency, some primer sets encompassed multiple gaps. PCR was conducted within a Veriti 96-Well Thermal Cycler (Thermo Fisher Scientific, Waltham, MA) in tubes with 50 µL of reaction mixture containing 1 µL of DNA template, 0.8 unit of Phusion DNA polymerase (Thermo Fisher Scientific), 10 mM deoxyribonucleotide triphosphates (dNTPs) mixture, 1× Phusion DNA polymerase buffer, and 10 µM of both forward and reverse primers. For fragments equal to or smaller than 3 kbp, PCR conditions comprised an initial denaturation at 98°C for 5 minutes, followed by 35 cycles of denaturation at 98°C for 45 seconds, annealing for 30 seconds, and extension at 72°C for 4 minutes, concluding with a final extension at 72°C for 10 minutes. In the case of fragments larger than 3 kbp, an alternative protocol was used, initiating with a denaturation step at 94°C for 2 minutes, followed by 40 cycles of denaturation at 94°C for 20 seconds, annealing for 45 seconds, extension at 68°C for 4 minutes, and a final extension at 72°C for 10 minutes. The amplified products underwent electrophoresis on a 1.0% (wt/vol) agarose gel in standard Tris/acetic acid/EDTA buffer and were subsequently purified using the Wizard Genomic DNA Purification Kit (Promega, Madison, WI). PCR fragments were sequenced by Sanger using both reverse and forward primers at Macrogen (Seoul, South Korea).

A region encompassing the *rrnB* operon was obtained using the specific primers rRNA2_F (5′-CGGATTCGCTCTAAGGGAGG) and rRNA2_R (5′-ACCTCGGTCCTTTCCACAAG). These primers were designed to match the unique regions both upstream and downstream of the *rrnB* operon. PCR conditions repeated those previously mentioned for fragments exceeding 3 kbp, with an annealing temperature set to 61°C. The resulting amplicon from CLas 9PA DNA was subsequently cloned into the pGEMT-Easy vector (Promega). The ligation mixture was employed for the transformation of competent *Escherichia coli* DH5α cells through electroporation (Bio-Rad, Hercules, CA). The cloned DNA underwent Sanger sequencing, and reads were analyzed using CodonCodeAligner.

### Genome assembly and annotation

Assembling took place within the CodonCodeAligner, using data from 9PA draft genome (JABDRZ000000000), along with reads obtained by PCR and Sanger sequencing after manual quality trimming. A minimum quality score of 20 was settled on while analyzing the reads. The ORFs within the 9PA genome were annotated using the PGAP (62) from the National Center for Biotechnology Information (NCBI), while the prophage region was annotated using the RAST Server (https://rast.nmpdr.org). In addition, prophage proteins and their functions were annotated using BLASTp non-redundant protein database (https://blast.ncbi.nlm.nih.gov/Blast.cgi). The 9PA and prophage maps were generated by CG view (http://cgview.ca/).

### Comparison of whole-genome organization of CLas strains

The complete genomic sequences available of CLas strains were downloaded from the NCBI (Table 2). Mauve software (63) was employed to perform whole-genome alignment and genomic structure comparison using the progressive Mauve approach, with the 9PA genome serving as the reference. Geneious Prime (version 2023.2.1; https://www.geneious.com/) was utilized to visualize data generated by Mauve.

**TABLE 2:** Genome of the strains of *Candidatus* Liberibacter asiaticus used in the whole-genome alignment

Strain	Origin	Size	CDS	Accession no.	Prophage type^*a*^
9PA	Brazil	1,231,218	1,025	CP145497.1	1 and 3
gxpsy	China	1,268,237	1,073	NC_020549.1	1, 2, and 4
A4	China	1,233,514	1,026	CP010804.2	2 and 4
JXGC	China	1,225,162	1,030	CP019958.1	3, 4
GDCZ	China	1,230,507	1,057	CP118922.1	2
PGD	China	1,230,220	1,030	CP100754.1	2
PYN	China	1,231,257	1,028	CP100417.1	1
CoFLP	Colombia	1,231,639	1,026	CP054558.1	1
ReuSP1	France (La Réunion)	1,230,064	1,023	CP061535.1	1
Ishi-1	Japan	1,190,853	982	AP014595.1	4
TaiYZ2	Thailand	1,230,623	1,028	CP041385.1	2
psy62	USA	1,227,328	1,021	NC_012985.3	1 and 4
JRPAMB1	USA	1,237,165	1,038	CP040636.1	2
AHCA1	USA	1,233,755	1,010	CP029348.1	1, 3, and 4

^*a*^ 4 denotes prophage-like sequence.

Subsequently, we proceeded to construct an NJ tree employing Molecular Evolutionary Genetics Analysis (version 11) (64). The comparison focused on the 14 sequences of CLas strains from NCBI database and included first, second, and third codon positions and non-coding regions. For the computation of genetic diversity between the compared genomes, the *p*-distance model was used, and the distance matrix was subjected to 1,000 randomizations to enhance its robustness. Through the comparison of the 14 genomes, HGRs were identified. In the selection of regions for investigation, we focused on those exhibiting high variability and occurrence frequency among the compared genomes, particularly when compared to the 9PA genome. Primers targeting the HGRs of CLas (Table 3) were designed using CodonCodeAligner.

**TABLE 3:** Primers used to access HGRs^*a*^ in *Candidatus* Liberibacter asiaticus strains

HGR	Primer set	Sequence	Temperature (°C)	Reference
HGR_2	2_F	TATCGTCCTCCCCCTTTTGC	61	This work
	2_R	GAGCATGGAGAATTCCTATCGA		
HGR_4	4_F	CGTCCTCTAAAAACTCCGTTGC	61	This work
	4_R	AGTAGGTTTACCGTGCGCTT		
HGR_6	6_F	TTGCGGGCTCTGCTTCTTC	61	This work
	6_R	ACGGTGGCTACAGCAGC		
HGR_7	7_F	ACGGTTTGCTTAAAGACGACA	61	This work
	7_R	GCCGTTACTTCTCTTACCGC		
HGR_12	12_F	AAGTGGGTCGGGTGGTTTTT	61	This work
	12_R	GTACGGTAGTTCACAGGCGA		
HGR_M	LJ1194f	TCAACAGAATACGCATCCAC	53	(46)
	LJ1195r	TCCTCATGAACGTGATTTGC		
	LJ240f	AAGAGCCTCATGCACATCAG	53	(46)
	LJ234r	CTGATATGTTAGTTCAAGCGG		

^*a*^ HGR, hypervariable genomic region.

### Sample collection for molecular screening

HLB symptomatic field samples of citrus were used for molecular screening of Brazilian CLas isolates. Between September and October 2022, 68 samples were collected across 12 localities within five areas characterized by medium and high incidences of HLB. These samples were distributed as follows: 37 from Avaré, 14 from Duartina, seven from Itapetininga, six from Limeira, and four from Matão (Fig. S4).

DNA was extracted using the CTAB method and analyzed by quantitative real-time (65) and conventional PCR to confirm CLas presence (66, 67) and to address amplification in gel electrophoresis. DNA samples of CLas strains maintained in greenhouses at Fundecitrus, São Paulo, Brazil (1PA, 2PA, 3A, and 9PA), and DNA samples of strains from Florida in the United States (F), Behai in China (B), Costa Rica (C), and Poona in India (I) were used as controls (27, 44).

### Molecular screening of CLas São Paulo population

For the screening of each HGR, PCR was conducted in a 20-µL mixture comprising DNA template at 100 ng/µL, 0.8 unit of Phusion DNA polymerase, and 0.2 mM dNTP mixture, 1× Phusion DNA polymerase buffer, and 0.2 µM of both forward and reverse primers. The reactions were set at an initial denaturation step to 98°C for 3 minutes, followed by 35 cycles of 98°C for 45 seconds, 55°C–63°C (depending on the primer set; see Table 3) for 30 seconds, a 72°C extension for 4 minutes, and a final elongation step at 72°C for 10 minutes. Agarose gel electrophoresis was run as indicated above.

Additionally, the presence of an ~8.3-kb region, previously detected in studies of CLas genome psy62 and described as a novel *Microviridae* phage (50), was assessed using PCR with specific primer pairs in the HGR_M (Table 3). The reaction mixtures and thermal cycling conditions followed the protocol outlined in Armstrong et al. (46). Agarose gel electrophoresis was run as indicated above.

### Prophage characterization

Alignments of 9PA prophages to SC1 (HQ377372, type 1), SC2 (HQ377373), and P-JXGC-3 (KY661963, type 3) were made using SnapGene software (Dotmatics, https://www.snapgene.com/) and BLAST global alignment. The integration sites in the 9PA chromosome were assessed by PCR amplifications based in previously described sites (7) using a set of specific primers (Table 4). PCR conditions were performed according to the protocol described in Zhang et al. (7), and the amplicons obtained were sequenced by Sanger.

**TABLE 4:** Primers used for prophage integration assessment in “*Candidatus* Liberibacter asiaticus“ 9PA chromosome

ID	Primer set	Sequence	Amplicon size (bp)		Temperature (°C)	Reference
A	G17F	TTGATCCTCGTCCTGTCCTT	2,608		59	(7)
	G17R	ATTAGTCCCAACATCTCAGT				
B	04,952F	TCATTTGTGGGGAGAGAAGT	2,616		59	(7)
	G17R	ATTAGTCCCAACATCTCAGT				
C	G17F	TTGATCCTCGTCCTGTCCTT	2,210		61	(7)
	33575RS	TCGGATCATAGGCTCAAACC				
G	G17F	TTGATCCTCGTCCTGTCCTT	2,832^*a*^	4,125^*b*^	61	(7)
	HP2_R	GGTATGGTGGAGTGTGAAAGAG				This work
H	HP32_F	ACCAGCAGATGAGCGAAATAG	2,446		59	This work
	33575RS	TCGGATCATAGGCTCAAACC				(7)
P	04,952F	TCATTTGTGGGGAGAGAAGT	2,218		61	(7)
	33575RS	TCGGATCATAGGCTCAAACC				

^*a*^ Referred to PCR amplicon for circular form of type 1 prophage.

^*b*^ Referred to PCR amplicon for circular form of type 3 prophage.

## Data Availability

The whole-genome sequence of 9PA strain generated in this study has been deposited in GenBank under the accession number CP145497.1. Additionally, the prophage genomes P-9PA-1 and P-9PA-3, respectively, have been deposited under the accession numbers PQ160487 and PQ160474. All data generated throughout this research have been cataloged in the Sequence Read Archive under the BioProject accession number PRJNA624158.

## SUPPLEMENTAL MATERIAL

Supplemental figures msphere.00376-24-s0001.docx Figures S1 to S4.

Supplemental Tables msphere.00376-24-s0002.docx Tables S1, S2, and S4.

Table S3 msphere.00376-24-s0003.xlsx Features of annotated ORFs in P-9PA-1 and P-9PA-3 sequences from *“Candidatus* Liberibacter asiaticus” 9PA from Brazil.

ASM does not own the copyrights to Supplemental Material that may be linked to, or accessed through, an article. The authors have granted ASM a non-exclusive, world-wide license to publish the Supplemental Material files. Please contact the corresponding author directly for reuse.
