# Supplementary material for: The complete genome sequence of “Candidatus Liberibacter asiaticus” strain 9PA and the characterization of field strains in the Brazilian citriculture
Source: mSphere. 2024 Nov 11;9(12):e00376-24. doi: 10.1128/msphere.00376-24 (PMC11656737; doi:10.1128/msphere.00376-24)
Supplement: Supplemental figures — Figures S1 to S4. [file msphere.00376-24-s0001.docx]

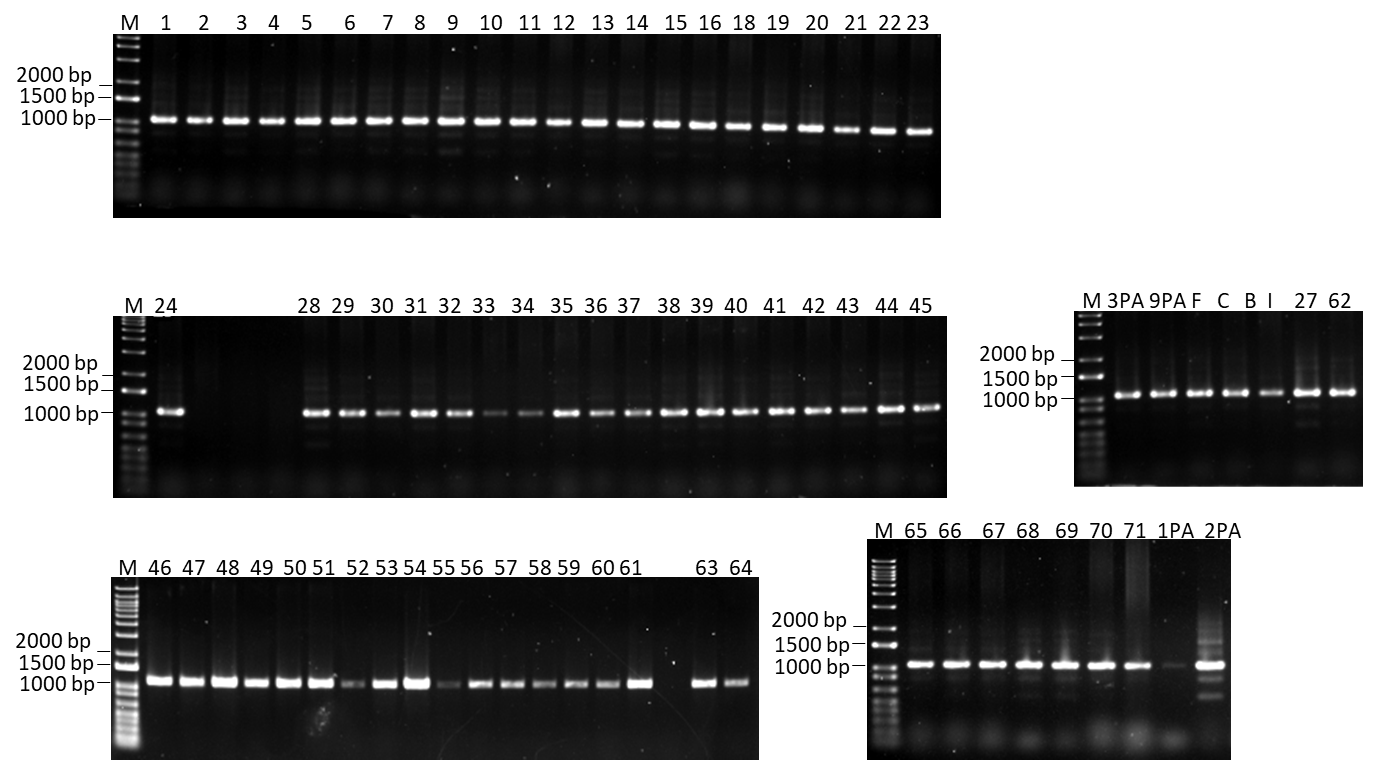


**Figure S1:** Amplicons of 1160 bp resulting from PCR to confirm presence of '*Candidatus* Liberibacter asiaticus' (CLas) in 68 field samples and 8 reference strains/controls. Results from PCR screening using primer set OI2c/ OI1 (Jagoueix et al. 1996). Lanes 1 to 16, 18 to 24 and 27 to 71 are DNA samples from field citrus trees; lanes 9PA, 1PA, 2PA and 3PA are DNA samples from Fundecitrus CLas strains kept in the greenhouse; lanes B – Behai, F – Florida, I – India and C- Costa Rica are control-DNA samples and lane M is 1-kb plus marker from Invitrogen.


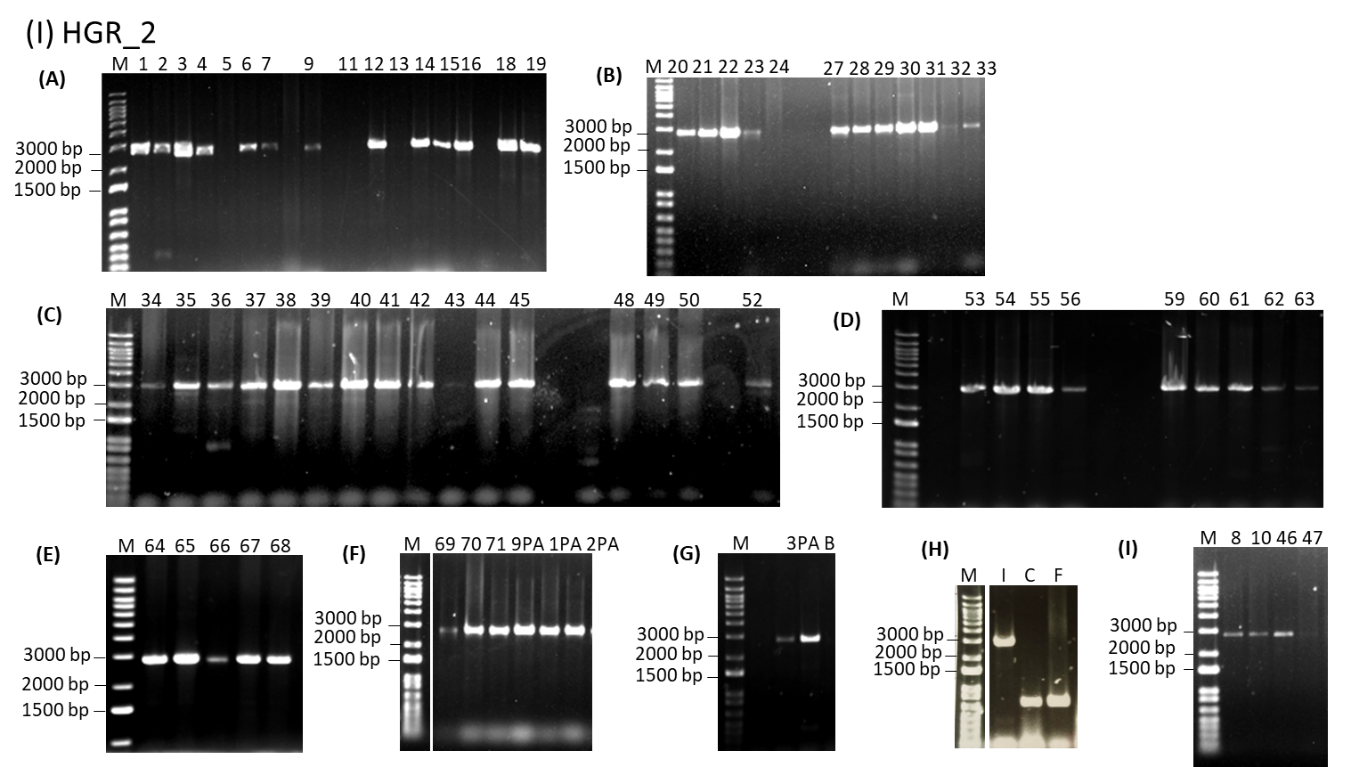


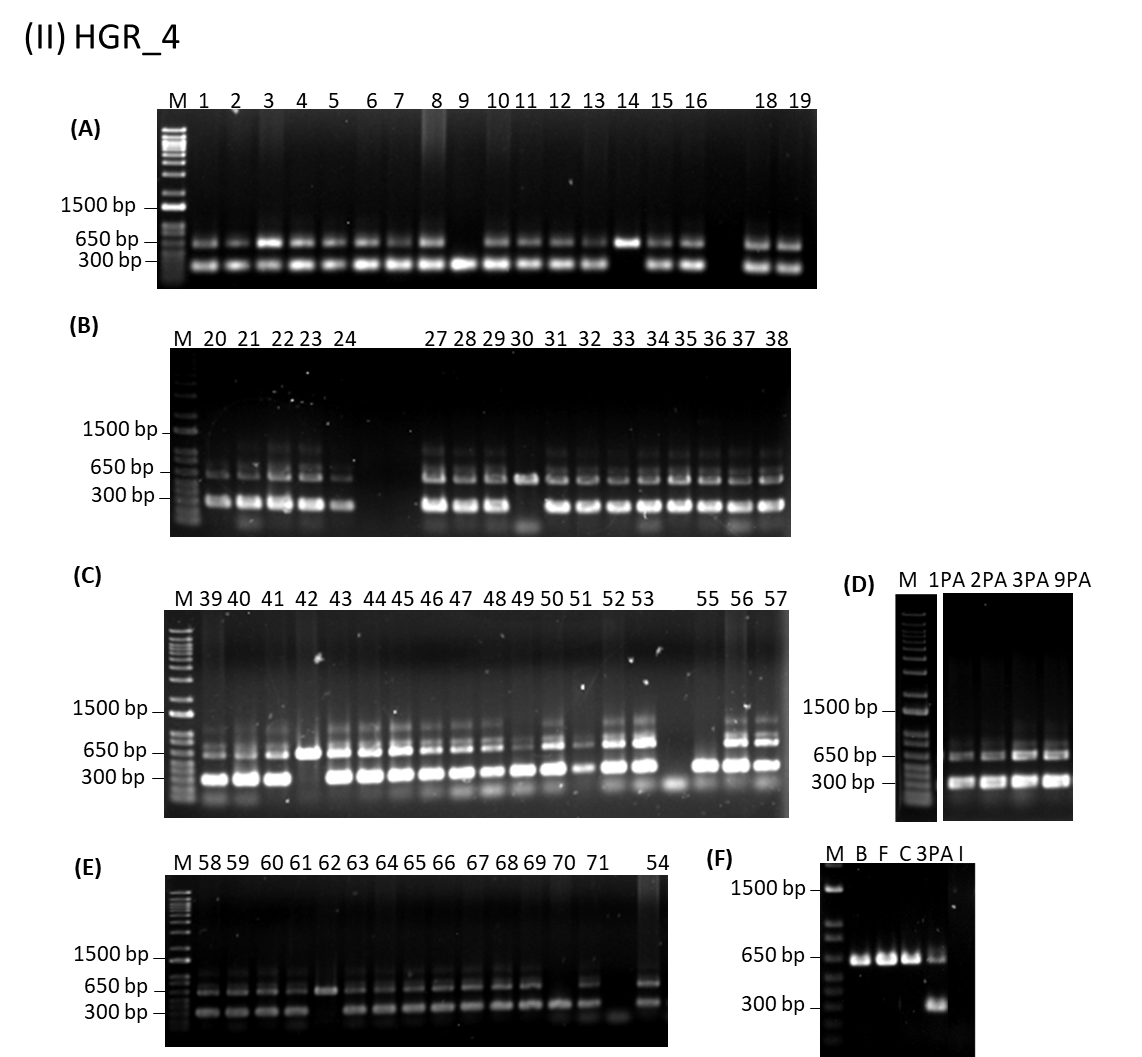


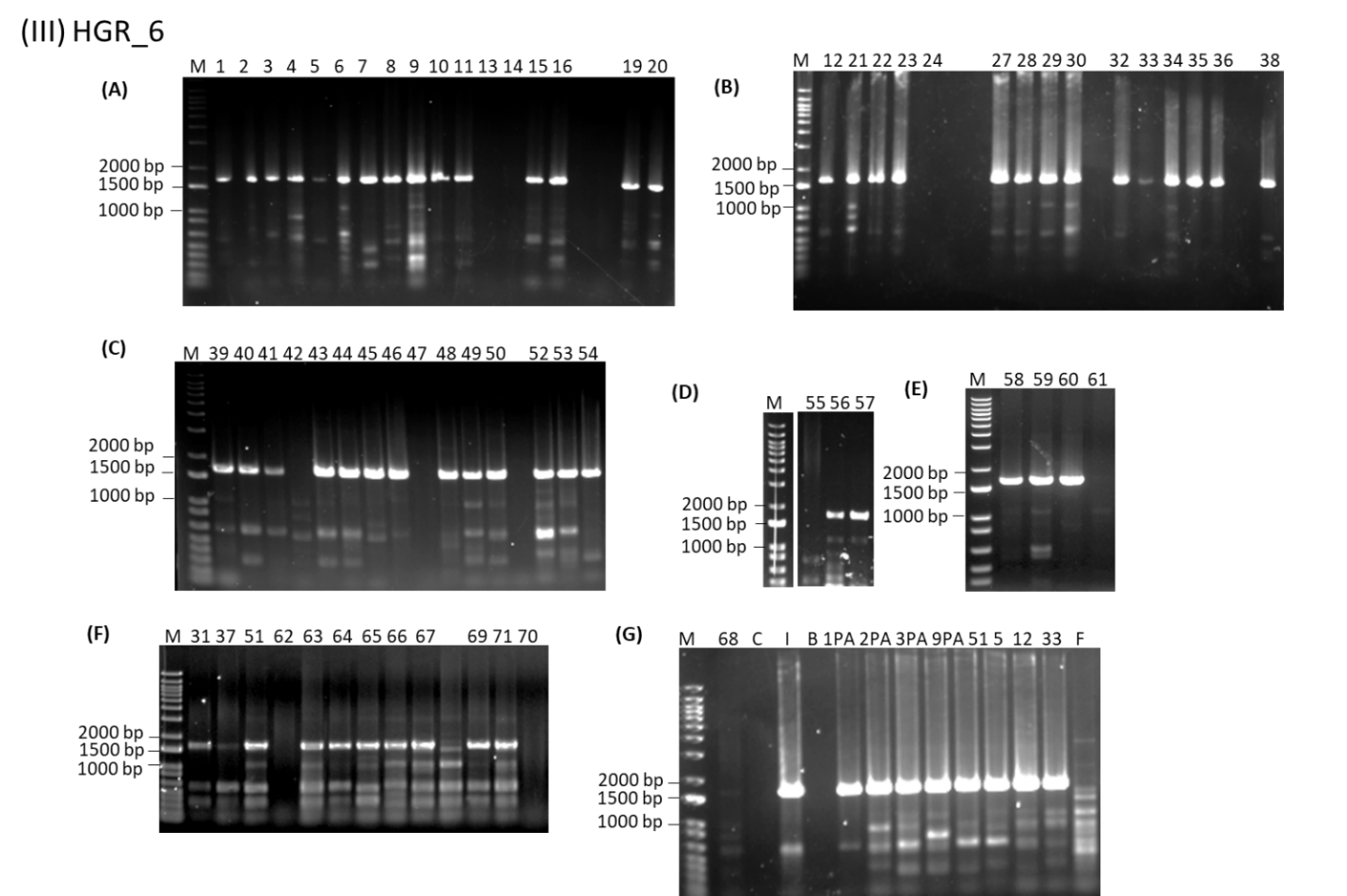


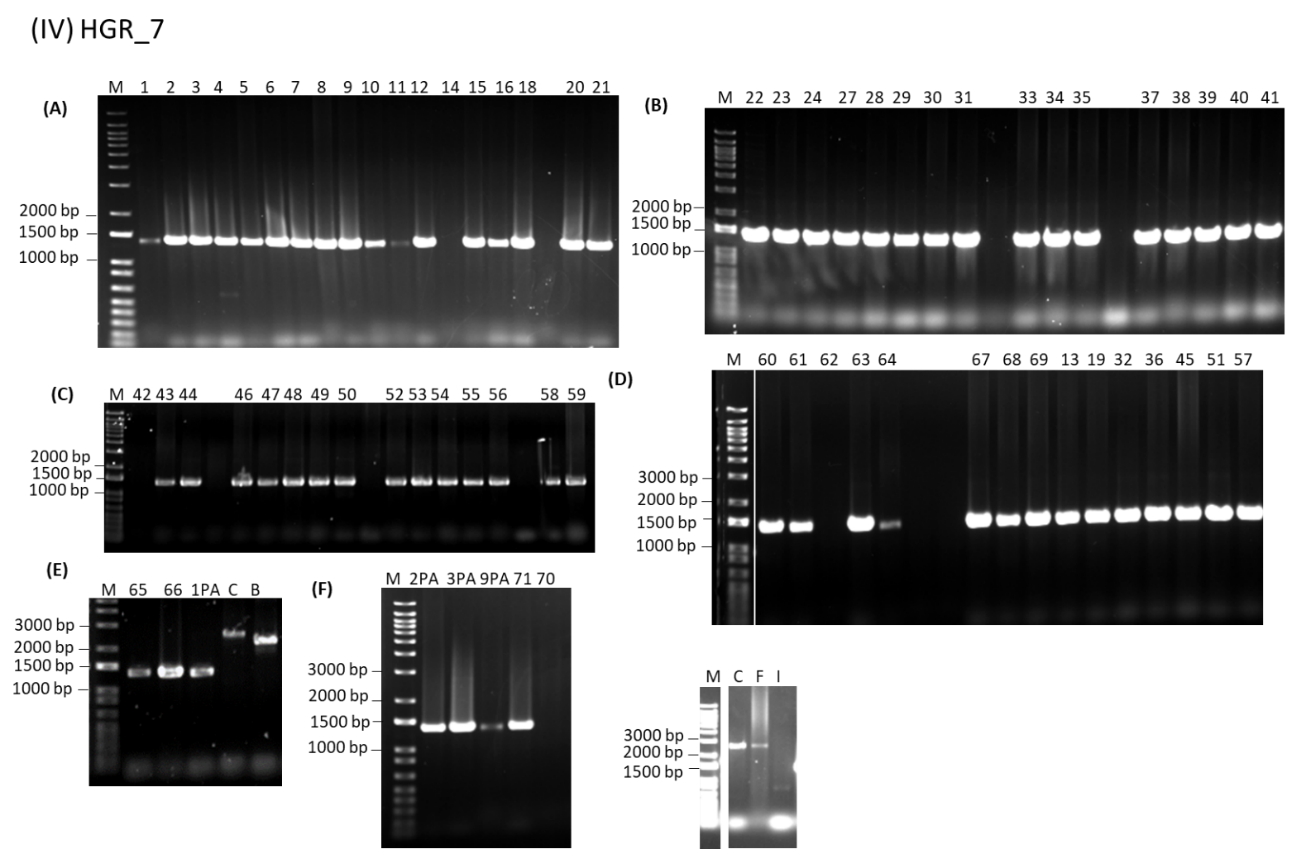


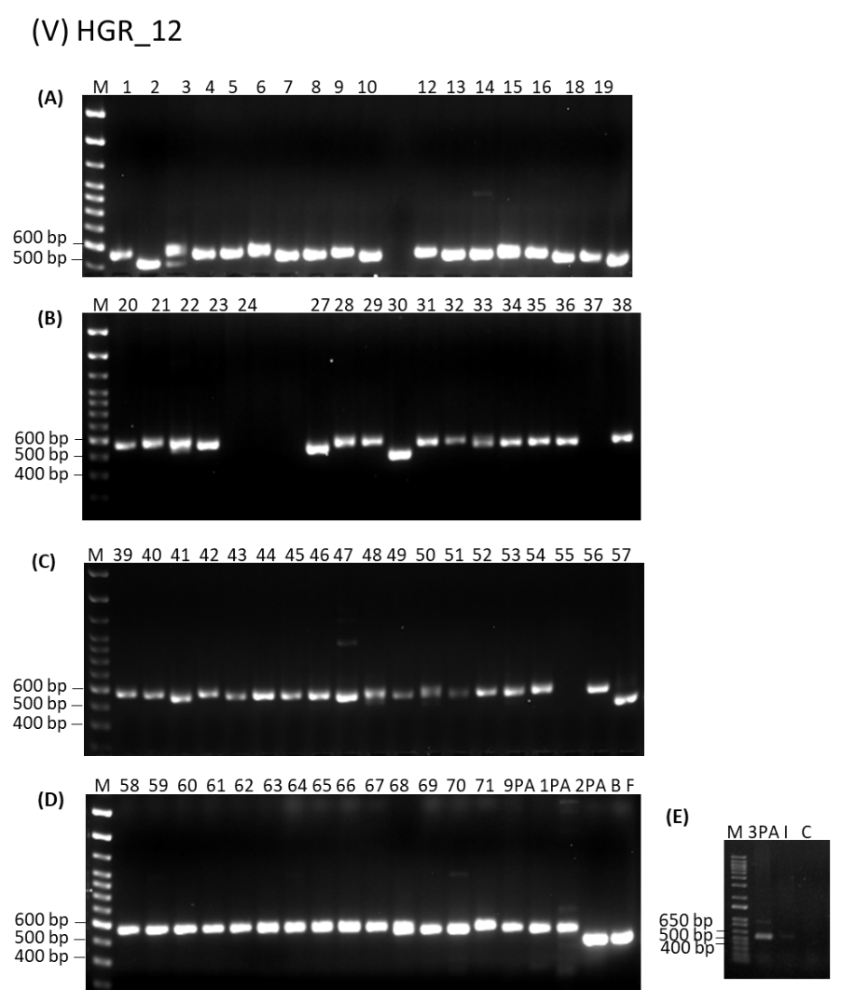


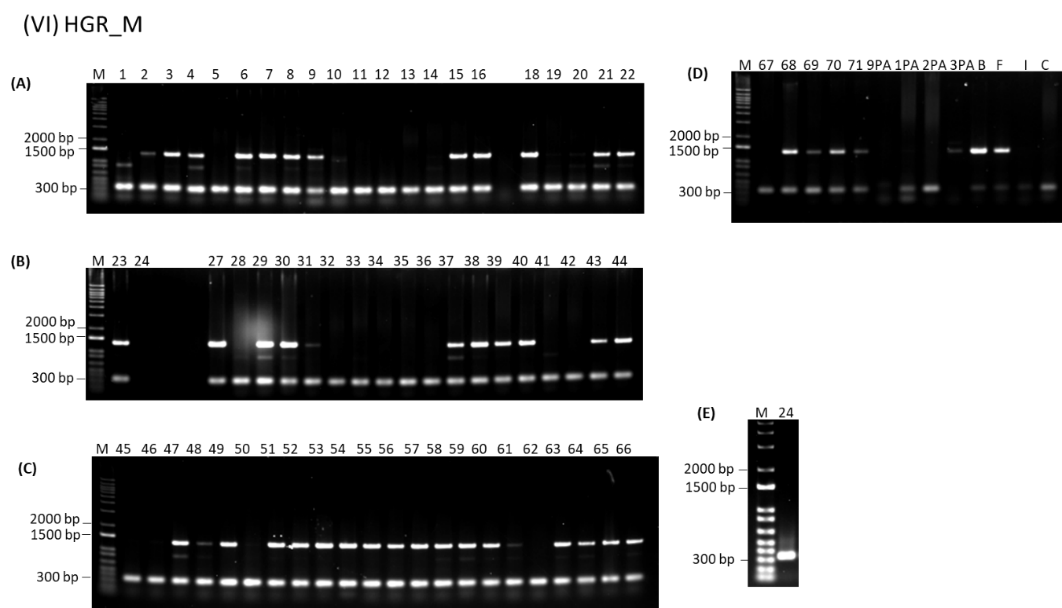


**Figure S2**: Amplicons generated from the screening of six highly hypervariable genomic regions (HGRs) in 68 field '*Candidatus* Liberibacter asiaticus' (CLas) strains and controls. Results from PCR screening for six HGRs using primers 2F/2R (I), 4F/4R (II), 6F/6R (III), 7F/7R (IV), 12F/ 12R (V) and LJ1194f/ LJ1195r and LJ240f/LJ234r (VI). In (I) to (VI) Lanes 1 to 16, 18 to 24 and 27 to 71 are DNA samples from field; lanes 9PA, 1PA, 2PA and 3PA are DNA samples from Fundecitrus CLas strains; lanes B – Behai, F – Florida, I – India and C- Costa Rica are control-DNA samples. In (I), (II) (III), (IV), and (VI) lane M is 1-kb plus marker from Invitrogen and in (V) and one gel of (II) lane M is 100 bp marker from Invitrogen.

**
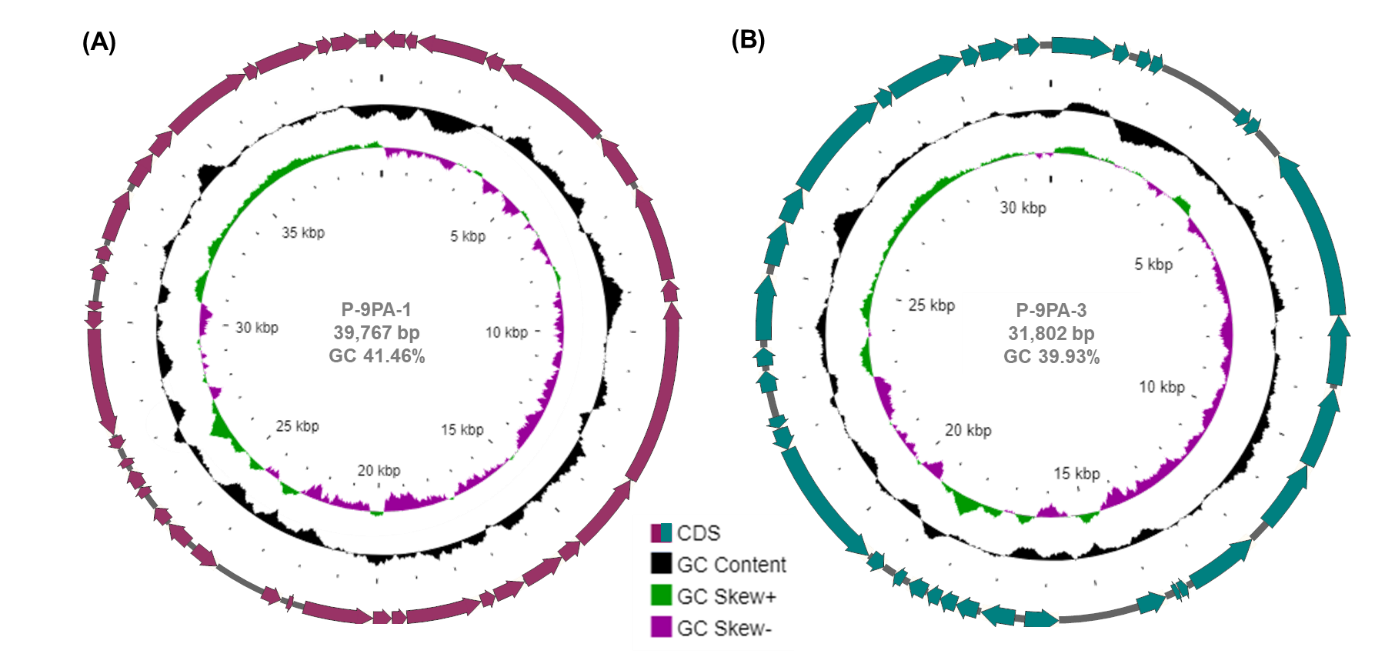
Figure S3:** Schematic representation of circular forms of prophages P-9PA-1 and P-9PA-3. In order from the outer ring to the inner ring showed coding sequences (CDS) of P-9PA-1 and P-9PA-3 genome (dark red and green, respectively), the GC content (black) and the GC skew [(G−C/(G+C))] (light green and purple).


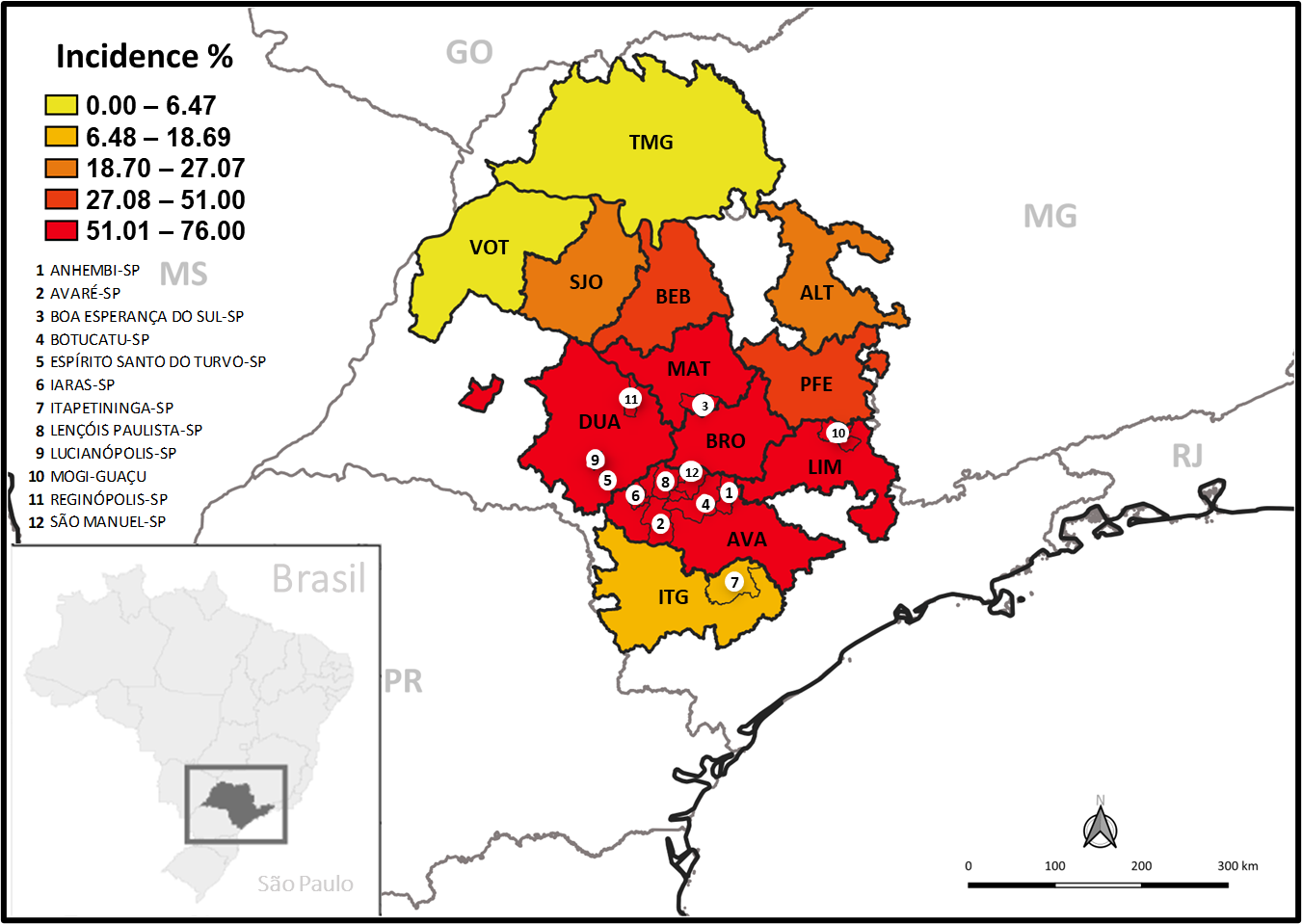


**Figure S4:** Map of citrus belt (São Paulo and Southwest of Minas Gerais states), illustrating the geographical locations where the *'Candidatus* Liberibacter asiaticus' samples were collected. The sampling efforts were concentrated in 12 localities within five regions characterized by both medium and high incidences of Huanglongbing, namely Itapetininga (ITG), Avaré (AVA), Duartina (DUA), Limeira (LIM), and Matão (MAT). Source: Fundecitrus ([www.fundecitrus.com.br](http://www.fundecitrus.com.br)).
