# Supplementary material for: The complete genome sequence of “Candidatus Liberibacter asiaticus” strain 9PA and the characterization of field strains in the Brazilian citriculture
Source: mSphere. 2024 Nov 11;9(12):e00376-24. doi: 10.1128/msphere.00376-24 (PMC11656737; doi:10.1128/msphere.00376-24)
Supplement: Supplemental Tables — Tables S1, S2, and S4. [file msphere.00376-24-s0002.docx]

Supplementary Table S1. Cycle threshold (Ct) values of samples used in molecular screening comparison of CLas strain from São Paulo fields.

| **Sample ID** | **Ct** | **Sample ID** | **Ct** |
| --- | --- | --- | --- |
| 1 | 24.6 | 42 | 23.3 |
| 2 | 23.4 | 43 | 23.1 |
| 3 | 24.6 | 44 | 23.7 |
| 4 | 23.9 | 45 | 22.7 |
| 5 | 23.7 | 46 | 23.2 |
| 6 | 23.8 | 47 | 23.4 |
| 7 | 24.4 | 48 | 26.3 |
| 8 | 22.7 | 49 | 25.1 |
| 9 | 24.0 | 50 | 24.2 |
| 10 | 24.5 | 51 | 24.2 |
| 11 | 24.4 | 52 | 25.4 |
| 12 | 23.8 | 53 | 24.3 |
| 13 | 24.2 | 54 | 25.0 |
| 14 | 24.1 | 55 | 24.4 |
| 15 | 24.0 | 56 | 25.1 |
| 16 | 24.2 | 57 | 25.2 |
| 18 | 23.9 | 58 | 24.1 |
| 19 | 23.5 | 59 | 25.2 |
| 20 | 24.0 | 60 | 24.4 |
| 21 | 25.1 | 61 | 24.7 |
| 22 | 24.2 | 62 | 24.5 |
| 23 | 23.8 | 63 | 25.1 |
| 24 | 25.5 | 64 | 25.5 |
| 27 | 22.8 | 65 | 24.6 |
| 28 | 24.8 | 66 | 24.2 |
| 29 | 24.8 | 67 | 24.2 |
| 30 | 24.3 | 68 | 24.5 |
| 31 | 25.3 | 69 | 26.4 |
| 32 | 25.3 | 70 | 26.5 |
| 33 | 25.9 | 71 | 26.3 |
| 34 | 25.1 | 9PA | 24.4 |
| 35 | 23.3 | 1PA | 23.31 |
| 36 | 24.5 | 2PA | 18.90 |
| 37 | 25.0 | 3PA | 29.21 |
| 38 | 23.0 | B | 22.93 |
| 39 | 25.3 | F | 24.72 |
| 40 | 24.2 | I | 21.69 |
| 41 | 25.9 | C | 24.13 |

Supplementary Table S2: Amplification results from 68 '*Candidatus* Liberibacter asiaticus' field strains (number from 1 to 71) and reference controls for six highly hypervariable genomic regions (HGRs).

| **ID** | **HGR_2** | | **HGR_4** | | | **HGR_6** | **HGR_7** | | | **HGR_M** | | **HGR_12** | | | **Profile** | **Region** |
| --- | --- | --- | --- | --- | --- | --- | --- | --- | --- | --- | --- | --- | --- | --- | --- | --- |
|  | **715** | **2822** | **292** | **601** | **624** | **1666** | **1400** | **2290** | **2593** | **330** | **1330** | **499** | **513/520** | **583** |  |  |
| 1 |  | + | + |  | + | + | + |  |  | + |  |  | + |  | A | Avaré |
| 12 |  | + | + |  | + | + | + |  |  | + |  |  | + |  | A | Duartina |
| 19 |  | + | + |  | + | + | + |  |  | + |  |  | + |  | A | Avaré |
| 20 |  | + | + |  | + | + | + |  |  | + |  |  | + |  | A | Avaré |
| 28 |  | + | + |  | + | + | + |  |  | + |  |  | + |  | A | Avaré |
| 32 |  | + | + |  | + | + | + |  |  | + |  |  | + |  | A | Itapetininga |
| 33 |  | + | + |  | + | + | + |  |  | + |  |  | + |  | A | Itapetininga |
| 36 |  | + | + |  | + | + | + |  |  | + |  |  | + |  | A | Itapetininga |
| 45 |  | + | + |  | + | + | + |  |  | + |  |  | + |  | A | Duartina |
| 46 |  | + | + |  | + | + | + |  |  | + |  |  | + |  | A | Duartina |
| 67 |  | + | + |  | + | + | + |  |  | + |  |  | + |  | A | Avaré |
| 9PA |  | + | + |  | + | + | + |  |  | + |  |  | + |  | A | Control, Brazil |
| 2PA |  | + | + |  | + | + | + |  |  | + |  |  | + |  | A | Control, Brazil |
| 34 |  | + | + |  | + | + | + |  |  | + |  |  | + |  | A | Itapetininga |
| 35 |  | + | + |  | + | + | + |  |  | + |  |  | + |  | A | Itapetininga |
| 1PA |  | + | + |  | + | + | + |  |  | + |  |  | + |  | A | Control, Brazil |
| 10 |  | + | + |  | + | + | + |  |  | + | + |  | + |  | B | Duartina |
| 2 |  | + | + |  | + | + | + |  |  | + | + |  | + |  | B | Avaré |
| 3 |  | + | + |  | + | + | + |  |  | + | + |  | + |  | B | Avaré |
| 4 |  | + | + |  | + | + | + |  |  | + | + |  | + |  | B | Avaré |
| 6 |  | + | + |  | + | + | + |  |  | + | + |  | + |  | B | Limeira |
| 7 |  | + | + |  | + | + | + |  |  | + | + |  | + |  | B | Limeira |
| 8 |  | + | + |  | + | + | + |  |  | + | + |  | + |  | B | Limeira |
| 15 |  | + | + |  | + | + | + |  |  | + | + |  | + |  | B | Duartina |
| 21 |  | + | + |  | + | + | + |  |  | + | + |  | + |  | B | Avaré |
| 22 |  | + | + |  | + | + | + |  |  | + | + |  | + |  | B | Avaré |
| 23 |  | + | + |  | + | + | + |  |  | + | + |  | + |  | B | Avaré |
| 27 |  | + | + |  | + | + | + |  |  | + | + |  | + |  | B | Avaré |
| 29 |  | + | + |  | + | + | + |  |  | + | + |  | + |  | B | Avaré |
| 31 |  | + | + |  | + | + | + |  |  | + | + |  | + |  | B | Itapetininga |
| 39 |  | + | + |  | + | + | + |  |  | + | + |  | + |  | B | Matão |
| 43 |  | + | + |  | + | + | + |  |  | + | + |  | + |  | B | Duartina |
| 51 |  | + | + |  | + | + | + |  |  | + | + |  | + |  | B | Avaré |
| 52 |  | + | + |  | + | + | + |  |  | + | + |  | + |  | B | Avaré |
| 56 |  | + | + |  | + | + | + |  |  | + | + |  | + |  | B | Avaré |
| 59 |  | + | + |  | + | + | + |  |  | + | + |  | + |  | B | Avaré |
| 60 |  | + | + |  | + | + | + |  |  | + | + |  | + |  | B | Avaré |
| 63 |  | + | + |  | + | + | + |  |  | + | + |  | + |  | B | Avaré |
| 64 |  | + | + |  | + | + | + |  |  | + | + |  | + |  | B | Avaré |
| 65 |  | + | + |  | + | + | + |  |  | + | + |  | + |  | B | Avaré |
| 66 |  | + | + |  | + | + | + |  |  | + | + |  | + |  | B | Avaré |
| 68 |  | + | + |  | + | + | + |  |  | + | + |  | + |  | B | Avaré |
| 48 |  | + | + |  | + | + | + |  |  | + | + |  | + |  | B | Avaré |
| 58 |  | + | + |  | + | + | + |  |  | + | + |  | + |  | B | Avaré |
| 69 |  | + | + |  | + | + | + |  |  | + | + |  | + |  | B | Avaré |
| 16 |  | + | + |  | + | + | + |  |  | + | + |  |  | + | C | Duartina |
| 18 |  | + | + |  | + | + | + |  |  | + | + |  |  | + | C | Avaré |
| **ID** | **HGR_2** | | **HGR_4** | | | **HGR_6** | **HGR_7** | | | **HGR_M** | | **HGR_12** | | | **Profile** | **Region** |
|  | **715** | **2822** | **292** | **601** | **624** | **1666** | **1400** | **2290** | **2593** | **330** | **1330** | **499** | **513/520** | **583** |  |  |
| 38 |  | + | + |  | + | + | + |  |  | + | + |  |  | + | C | Limeira |
| 44 |  | + | + |  | + | + | + |  |  | + | + |  |  | + | C | Duartina |
| 54 |  | + | + |  | + | + | + |  |  | + | + |  |  | + | C | Avaré |
| 71 |  | + | + |  | + | + | + |  |  | + | + |  |  | + | C | Duartina |
| 53 |  | + | + |  | + | + | + |  |  | + | + |  |  | + | C | Avaré |
| 9 |  | + | + |  |  | + | + |  |  | + | + |  | + |  | D | Limeira |
| 13 |  |  | + |  | + |  | + |  |  | + |  |  | + |  | E | Duartina |
| 5 |  |  | + |  | + | + | + |  |  | + |  |  | + |  | F | Avaré |
| 11 |  |  | + |  | + | + | + |  |  | + |  |  | + |  | F | Duartina |
| 14 |  | + |  |  | + |  |  |  |  | + |  |  | + |  | G | Duartina |
| 62 |  | + |  |  | + |  |  |  |  | + |  |  | + |  | G | Avaré |
| 42 |  | + |  |  | + |  |  |  |  | + |  |  | + |  | G | Matão |
| 30 |  | + |  |  | + | + | + |  |  | + | + | + |  |  | H | Itapetininga |
| 40 |  | + | + |  | + | + | + |  |  | + | + | + |  |  | I | Matão |
| 57 |  | + | + |  | + | + | + |  |  | + | + | + |  |  | I | Avaré |
| 61 |  | + | + |  | + |  | + |  |  | + | + |  | + |  | J | Avaré |
| 47 |  | + | + |  | + |  | + |  |  | + | + |  |  | + | K | Duartina |
| 55 |  | + | + |  |  |  | + |  |  | + | + |  | + |  | L | Avaré |
| 24 |  |  | + |  | + |  | + |  |  | + |  |  |  |  | M | Avaré |
| 70 |  | + | + |  |  |  |  |  |  | + | + |  | + |  | N | Duartina |
| 37 |  | + | + |  | + | + | + |  |  | + | + |  |  |  | O | Limeira |
| 49 |  | + | + |  | + | + | + |  |  | + | + |  |  |  | O | Avaré |
| 41 |  | + | + |  | + | + | + |  |  | + |  |  |  | + | P | Matão |
| 50 |  | + | + |  | + | + | + |  |  | + |  |  |  | + | P | Avaré |
| USA (Florida) | + |  |  | + |  |  |  |  | + | + | + | + |  |  | Q | Control |
| Costa Rica | + |  |  | + |  |  |  |  | + | + |  |  |  |  | R | Control |
| India |  | + |  |  |  | + |  |  |  | + |  |  | + |  | S | Control |
| China (Behai) |  | + |  |  | + |  |  | + |  | + | + | + |  |  | T | Control |
| 3PA |  | + | + |  | + | + | + |  |  |  | + |  | + |  | U | Control, Brazil |

+ = presence of PCR product.

Supplementary Table S4. Primer sets designed and used for 9PA genome gap closure using PCR.

| Primer | Sequence | Amplicon size (bp) | Gap code |
| --- | --- | --- | --- |
| 42f_9PA | AATCCGTGCGATAGCATTTC | 2710 | 1 |
| 44r_9PA | GGAATGCCAAATTTATCTTCTTTT |  |  |
| 44f_9PA | TACCAGCTGCTGCGATCATA | 1252 | 2A |
| 45r_9PA | TTTTTCAGTTCCACGCAAGA |  |  |
| 45f_9PA | GGCAATATGCCTGCTTTCAT | 447 | 2B |
| 46r_9PA | TTCTCCTATCGCGAGCATTT |  |  |
| 46f_9PA | AACCCTAGCTGCGACCATAA | 1994 | 3 |
| 47r_9PA | GCTTTAAAACAAGCGGCTCA |  |  |
| 47f_9PA | CGCCCTCCTCAAAATAACAC | 414 | 4A |
| 48r_9PA | TGATTTGCATTCCCTCGATT |  |  |
| 48f_9PA | TCTATTTTGCGGTGTATGCAA | 392 | 4B |
| 49r_9PA | GGTACCTGATGACTTCACACGA |  |  |
| 49f_9PA | TCTGTAAAGCAAATAGCGCTTAATA | 4752 | 5 |
| 53r_9PA | GGATCAACGAGTTGCAAAAA |  |  |
| 53f_9PA | TCGAAACCATATTACTTGAATAGGAA | 406 | 6 |
| 54r_9PA | TGTTGCTGCAGGATTAGCTG |  |  |
| 54f_9PA | GAAGCACCTGAGGAACAAGC | 420 | 7 |
| 55r_9PA | TTTTGACACCCCACAAGTCA |  |  |
| 55f_9PA | GCAAAACCAATGGAAGACGCT | 2150 | 8 |
| 56r_9PA | TTACCCATCCATCAGCAGCC |  |  |
| 56f_9PA | GACCTGTTCACTATATCCCGAAG | 4878 | 9 |
| 58r_9PA | TGGAATAGATGGCACGCTAA |  |  |
| 58f_9PA | AGCTGAGCTATCTGGGCATT | 492 | 10 |
| 59r_9PA | CGCATATCACTTGGGGATAAA |  |  |
| 59f_9PA | CACCCAACAGCTAGCACTCA | 2858 | 11 |
| 60r_9PA | CTGGTGATGGGCAGGTAAGT |  |  |
| 60f_9PA | TGAAGGTGATTCTGCTGGTG | 872 | 12A |
| 61r_9PA | GAACAAGGGCCTGTTATTTTT |  |  |
| 61f_9PA | TGAAATTTTTCCTTTCGGATTC | 431 | 12B |
| 62r_9PA | CGCGAAATTTTCTCATCACA |  |  |
| 62f_9PA | TTCATGTGCACGGTGGTATT | 1314 | 13 |
| 63r_9PA | AACACGTCCCATCAAACCAT |  |  |
| 63f_9PA | TCAGAGTTTGGAAGAGCTTTGAG | 5498 | 14 |
| 65r_9PA | TTCCAAATTCATTGGCGATT |  |  |
| 65f_9PA | TGATTCTTTGGGTGCATTGA | 1243 | 15A |
| 66r_9PA | CTATCCGTTCGGTGCAAACT |  |  |
| 66f2_9PA | TGATGGAGCTGTTTTGGCAA | 2525 | 15B2 |
| 68r2_9PA | ACAGGAAGACACATGCACCT |  |  |

| 68f_9PA | GGGGTCATTGTAGCAAAACG | 1826 | 16 |
| --- | --- | --- | --- |
| 70r_9PA | ACAATGCCAAAACATCATGC |  |  |
| 70f_9PA | GAGTCCGTTGATGTTTTGGTT | 2186 | 17 |
| 72r_9PA | TCCACTATCCTCTTTGCAAAATC |  |  |
| 72f_9PA | TGTGGCATTCCTGTTGTTTCT | 1266 | 18 |
| 73r_9PA | CGGTTGAACCATGTGCTATG |  |  |
| 73f_9PA | TCAGCGAAGTATTCACTTCCAA | 1135 | 19A |
| 74r_9PA | ATAGCAAGTGGGAAGACGCG |  |  |
| 74f_9PA | GGATTGAATGCCTAGGAGAGAG | 3807 | 19B |
| 77r_9PA | CTTTAGACGAAACGCGATCA |  |  |
| 77f_9PA | ACTGCTGAAGGTGCAAGGTT | 2985 | 20 |
| 78r_9PA | GAGCAGCATGAAGAAATGGCT |  |  |
| 78f_9PA | TACGCGCTATTGTTGCAAGT | 1881 | 21A |
| 79r_9PA | AAGTGGCGCAAAGAACAATC |  |  |
| 79f2_9PA | ACACACTGCCATTCCCAATACA | 986 | 21B |
| 80r2_9PA | ACAGGTATTATTCTCACCCGCA |  |  |
| 80f_9PA | TCTTGATCCTGATACGAAATTCAA | 3685 | 22 |
| 82r_9PA | TCTCCGACAACTGTTCTAACGA |  |  |
| 82f_9PA | TCGCTAAATCCCGTTTTCTC | 505 | 23 |
| 83r_9PA | TCCTTCAGCAATTCCAGGAC |  |  |
| 83f_9PA | CAGGACAAATGATGATGCGACA | 5478 | 24 |
| 84r_9PA | TGCACCGCAAATACAGCAAA |  |  |
| 84f_9PA | GAACACACTTCATGTTCTTCCTTTT | 5415 | 25 |
| 86r_9PA | GCGAGCTAAACAACGAAAGC |  |  |
| 86f_9PA | ACGATGTCCATCCATTTTCC | 1477 | 26 |
| 88r_9PA | CTGGCGTTACACGATCAAAA |  |  |
| 88f_9PA | AGGGGGTTCATGAAAACTCTCC | 1425 | 27 |
| 89r_9PA | TTTAATTTCCTCACTCTTCGTTG |  |  |
| 89f_9PA | AGAGCACGTTTTTGAGGATCA | 1826 | 28 |
| 91r_9PA | GGAGAAACCGTTAGCAAAGC |  |  |
| 91f_9PA | CCATCTGGTCCCCAAGCTTT | 4322 | 36 |
| 94r_9PA | AAGGCTCCTGAGACAGTCGT |  |  |
| 94f_9PA | AGCATTACTAGTGCACATGGGT | 3954 | 37 |
| 96r_9PA | TGCTCATTGAACTTGAGGGGA |  |  |
| 96f_9PA | AGTGCGCAATCGTTCTAGCA | 1474 | 38A |
| 97r_9PA | CGTGCCACTAATCCCATGGG |  |  |
| 97f_9PA | ATGAAGGATCGGCGATCGGT | 2322 | 38B |
| 175r2_m9PA | TCCCTCATATCCTTTTCTGCCT |  |  |
| 175f_m9PA | AGATTTCGTTCAAGCACATGGT | 2042 | 38C |
| 98r_9PA | TCGCCTAACTCAGAAGGAAGT |  |  |
| 175f_m9PA | AGATTTCGTTCAAGCACATGGT | 1907 | 39 |
| 98r_9PA | TCGCCTAACTCAGAAGGAAGT |  |  |

| 98f_9PA | TGCTGTTGTAGATTTGCGACG | 477 | 40 |
| --- | --- | --- | --- |
| 99r_9PA | ACGACAAATAGAAGCACGTGC |  |  |
| 99f_9PA | CCTCGTCCTTATTGGGTGTCA | 3440 | 41 |
| 101r_9PA | GGCCCTGCTTGTGAAAATCA |  |  |
| 101f_9PA | GGATAGCTTGGCAATCTATCGG | 3278 | 42 |
| 103r_9PA | GGATACCGCCCCTATTGTCA |  |  |
| 103f2_9PA | GGTCTTCCAGCAATGTGAAGGT | 3302 | 43A |
| 190r_m9PA | AGACCACAAACACTGCGCAA |  |  |
| 105r_9PA | ACTACCGTCTTGTTTGGTGTTT | 1414 | 43B |
| 190f_m9PA | CTGCCCGTCCAGTCTCTACA |  |  |
| 105f_9PA | TGTGTGAACAAAGAACAGGCG | 3457 | 44 |
| 107r_9PA | GAAAGACGAAACGCTTGCGA |  |  |
| 107f_9PA | GCCACGTGATGTTACCTTGC | 4838 | 45 |
| 109r_9PA | TCCTTCTTTTTGTGACCGAACT |  |  |
| 109f_9PA | TGCCAAATGCTAATGACGATGT | 569 | 46A |
| 110r_9PA | AACGTCCCATCAGAACCG |  |  |
| 110f2_9PA | GTAGCTATAGATGATACACC | 897 | 46B |
| 111r_9PA | GGCGATGATGTGCAAAACCT |  |  |
| 111f_9PA | TGGCGACTATACAGCTCTATGA | 4328 | 47 |
| 113r_9PA | CGTGCTGATTATGACCAGGGA |  |  |
| 113f_9PA | GGGCGTTGTTTGCATGAGTT | 5732 | 48 |
| 116r_9PA | GCCAGATCTAGCGGGAATTCC |  |  |
| 115f_9PA | TCCCAAGGGCTAATAACTTGCA | 2708 | 49 |
| 117r_9PA | TGAATCAGCATCACGAAGACAA |  |  |
| 49_1f_9PA | CCCGATCGGATTTGGTAAA | 1500 | 49-1 |
| 49_1r_9PA | GACTCTATCCAGAATCTTTATGTTC |  |  |
| 49_1f_9PA | CGTAGAGATGCAACGGCTATC | 1500 | 49-2 |
| 49_1r_9PA | TCCTTTAGTGCCGTGAGATTG |  |  |
| 119f_9PA | ACGAATTGCGAGAGAACGGT | 3061 | 50 |
| 121r_9PA | ACCTAGCCTCTCGAAAACGA |  |  |
| 120f_9PA | ACACCTTTCCCAACTGTTCCT | 5061 | 51 |
| 123r_9PA | CTGAAACAACTGCTGAAGACGA |  |  |
| 123f_9PA | GTCAATTTGTTCCGTGATTGCA | 4250 | 52 |
| 125r_9PA | AACCAACTGAGCTACACCCG |  |  |
| 125f_9PA | TTGTTGGGGGCAGATGAGTT | 638 | 53A |
| 126r_9PA | TGCGATCCATAAGCAAAACGT |  |  |
| 126f_9PA | GCAATATGCGTATCCTCTCGAA | 3203 | 53B |
| 127r_9PA | GCACATGAGCATTGGGTAGC |  |  |
| 127f_9PA | TGCAACCGCGTTTGTGAATT | 5978 | 54 |
| 235r_m9PA | TCACACGCTTCAATGGTCGA |  |  |
| 54_1f_9PA | TGTTCGTATTCAACTGGGTAGAG | 1500 | 54-1 |
| 54_1r_9PA | CTTAGCAAGCAGCGTGAATTT |  |  |

| 54_2f_9PA | TCTGGTGGACCTGTTGTTATG | 1630 | 54-2 |
| --- | --- | --- | --- |
| 54_2r_9PA | ACCATTCGCCAACTATCCTATAC |  |  |
| 130f2_9PA | GCAATTTAGCGCGATTTCCTGA | 1013 | 55A2 |
| 131r_9PA | CCGTAAAAACACAACTCTCACC |  |  |
| 131f3_9PA | GAGTTGAAATTTTTGCTACGCG | 1299 | 55B2 |
| 133r_9PA | TGACCACTACAGGCAGAGGA |  |  |
| 134r_9PA | TGCTGAAAACTTAGAGCGTGT | 1141 | 56A |
| 133f_9PA | TCTTCTCCCATAACAGCCGAGA |  |  |
| 134f2_9PA | ATATTGGTATGGTAGCGCGTG | 1299 | 56B2 |
| 135r2_9PA | AGTCCGTTTACCCTCTTTTTCA |  |  |
| 135f2_9PA | CCCATTGCAAATACTGGAGCA | 1467 | 57A |
| 136r_9PA | TTCATTTGGACGGCGAACAC |  |  |
| 136f2_9PA | TGGTTGAAAATCGGCAATGAAG | 3069 | 57B2 |
| 138r2_9PA | GCGTATCTTGATCCGGAGAAAA |  |  |
| 138f_9PA | GTAAAGGGGCAAGGCGATCC | 1620 | 58A2 |
| 139r3_9PA | ACGCGCAATGTTCATTTTCTT |  |  |
| 139f_9PA | ATACAGTAAGATATAACATA | 854 | 58B |
| 140r_9PA | TCGTCTTCCAAGAAGCATGCC |  |  |
| 140f_9PA | TGTGAGGTGCATTGCTGGTC | 2364 | 59 |
| 142r_9PA | GTCAATGGCCTAAGACGCGT |  |  |
| 142f_9PA | TGGTGGTTATGTAGATGCTCGT | 2068 | 60 |
| 259r_m9PA | AGCTGTGGGGATTAAACGCA |  |  |
| 259f_m9PA | GCGTGCTAAAGGGGCAGTAA | 462 | 61A |
| 261r_m9PA | AACGTACAATATGCTTGCCGGA |  |  |
| 259f3_m9PA | AGCCGACATTATCCACATCACA | 1228 | 61B2 |
| 144r2_9PA | ACCACGCGTGCACTTTTT |  |  |
| 144f2_9PA | AGTTCCACGGCATGTTGAAG | 2440 | 61C2 |
| 145r_9PA | CCTCGTGGCGGAATGGTTAC |  |  |
| 145f_9PA | TGAGCATGCATCGTCTCAGT | 1408 | 62A |
| 146r2_9PA | ACCAGCATGATCCGTTCCTG |  |  |
| 146f_9PA | GAGGCGTATCAAAAGTGCATGT | 4096 | 63 |
| 147r_9PA | CACGTCCAAATCCCCTCACG |  |  |
| 147f_9PA | CCTTTGGCAGCATGTAAAGTCA | 2200 | 64A |
| 148r_9PA | AAAGTTGTGATTTCGGCGGA |  |  |
| 148f_9PA | CACTGACTAGGCAGCGAGAA | 1520 | 64B |
| 277r_m9PA | ACAGACGACTCCATAACGTCA |  |  |
| 278r_m9PA | AACGATCCTCATCCTGCTCT | 815 | 65A |
| 277f_m9PA | TGACGTTATGGAGTCGTCTGT |  |  |
| 279r_m9PA | GATGGTCGTGGTCTCTTTGCA | 397 | 65B |
| 278f_m9PA | TGCGCAGTGAGAATTTCGAG |  |  |
| 279f_m9PA | TGCAAAGAGACCACGACCATC | 3755 | 66 |
| 152r_9PA | CACCAGTTGCTATCCTCGAGG |  |  |

| 152f_9PA | GCACGAATAGCAACAAAACCCA | 3350 | 67 |
| --- | --- | --- | --- |
| 153r_9PA | GCGACAATTCTCAATGCAAGCA |  |  |
| 153f2_9PA | ACTCAAGACATCATCGCGTGT | 1493 | 68 |
| 154r2_9PA | AGTACTGATCTCCTCCTTGTGG |  |  |
| 154f_9PA | CACTTAATCGCTTTCCTCTGGA | 779 | 69A |
| 155r_9PA | TGTCTGAACAGGAAGGAGTCT |  |  |
| 155f_9PA | TCAAGTTTTTTAGGATGATT | 2494 | 69B2 |
| 289r3_m9PA | ATACCAAACTAGCGCATGAAGT |  |  |
| 289f_m9PA | TCGCCTTGGCAGCATAGAGA | 4735 | 70 |
| 158r_9PA | CTTCCTGTGGTTGTGCTGGT |  |  |
| 158f_9PA | TCGTTCTTGCCCAATACCTACA | 4391 | 71 |
| 160r_9PA | CGCCTCCGCCTTATTCTTCA |  |  |
| 160f_9PA | TCAAGGAGGTGATGGTCCCC | 1246 | 72A |
| 161r_9PA | TCTGCTCAATCAAGTATGCCGA |  |  |
| 161f_9PA | CCAGGCGGTCCAATCATGATC | 4072 | 72B |
| 162r_9PA | GTTGTTACATAGTGGCAGTGCA |  |  |
| 162f_9PA | GGTCCTATGGTTCTTGATGGGC | 3918 | 73 |
| 164r_9PA | ACCACACCCTTAGCAACTGG |  |  |
| 164f_9PA | TGGTGAATCAGTTGAAATCGGT | 870 | 74A |
| 165r_9PA | ACCAAGACGCGCAATCTCTCTT |  |  |
| 165f_9PA | TGGTTCCCACTGCGGTTTTT | 961 | 74B |
| 166r_9PA | GGGAAAAAGCCGCTGAGGAA |  |  |
| 166f_9PA | GCCGCACATCCTTCTCCATT | 1042 | 75A |
| 167r_9PA | ATATTCCCTGCGAGCGATCTGA |  |  |
| 167f_9PA | TGATTGTCTCTCTGGGTTGCT | 446 | 75B |
| 168r_9PA | ACGCGATTACGTCCTGATTTT |  |  |
| 168f2_9PA | ACGACGACATTCAGGATCGAGT | 961 | 76 |
| 169r_9PA | ATGCCCGGTTGGTGGAATTG |  |  |
| 169f2_9PA | TCCCGCAAGCATTTCAGAGC | 866 | 77 |
| 170r2_9PA | TGTCCCTAAAAGCATTGCTCTT |  |  |
| 170f_9PA | GGCATACGTGTTGTGTTTGACA | 2151 | 78A |
| 171r_9PA | TTGTGTCGACCAGTGAGTCA |  |  |
| 171f_9PA | TGCATTGAACCAGAAAGATCCA | 3156 | 78B |
| 173r_9PA | CGCAACGAAGGAATCAAAGACG |  |  |
| 171f_9PA | TGCATTGAACCAGAAAGATCCA | 3421 | 78B2 |
| 173r2_9PA | CGATTCGCTGTGCTGTTTGAA |  |  |
| 173f_9PA | TGCCAACATATCACCTAGCAAA | 4709 | 79 |
| 175r_9PA | TCCGCCAAATCTAAACGTGGA |  |  |
| 175f2_9PA | CGCAAGGCACGATTGATCTT | 2294 | 80A |
| 176r_9PA | TGCTGAGCTCAAAGAGGGAT |  |  |
| 176f_9PA | ACAAGGTTTGTTGCGATTGCA | 2837 | 80B |
| 177r_9PA | GATCGGGGCGTTGGAAAGTA |  |  |

| 177f_9PA | GTAAGCGGGGACATTCAGCA | 2902 | 81 |
| --- | --- | --- | --- |
| 179r_9PA | CGTGCGGCATTGATCACTTC |  |  |
| 179f_9PA | ACATTTGCCTGTACTTGACGTG | 1912 | 82A |
| 325r_m9PA | TGGCACGACAATTGCGTGTA |  |  |
| 325f_m9PA | TGATCCGATGCGAGGTCACA | 3637 | 82B |
| 180r_9PA | GCAAGAATGGCACCAGGGAT |  |  |
| 181r2_9PA | AGGAATGGGTAAATGGTAGGGA | 2427 | 83A2 |
| 180f3_9PA | ATCTCATGGCTTCCTCGACA |  |  |
| 181f_9PA | ACCAGTCCATCCTCTCCGAAAT | 721 | 83B |
| 182r_9PA | TCTTTTCACCGATTCCTGGGA |  |  |
| 182f_9PA | GCGGGCAAAGTGAAGACGAT | 3526 | 84 |
| 183r_9PA | TCCTCTTGAAGATTGGAAGCGT |  |  |
| 185f_9PA | TCTTCGTCTGTTGTTTCCCCA | 1935 | 88 |
| 186r3_9PA | ATTGGGTGTTGTTCTCGGCT |  |  |
| 1f_9PA | TCCCTTTCAACCTAGATCCTGA | 1706 | 89 |
| 2r_9PA | AGACAATGTGCAAGTGATCCA |  |  |
| 2f_9PA | TTGCTGGACGTTCTGGAAGT | 2085 | 90 |
| 3r_9PA | TGCGTTGTTTCTCTTACGTCA |  |  |
| 3f_9PA | GCGGGATCAGTAATTTCAACC | 2083 | 91 |
| 4r_9PA | ACTCCAGGGCGCATGATTAT |  |  |
| 4f_9PA | TGAGAAAGCTGCGAGGAACA | 2545 | 92 |
| 5r_9PA | AGGGAATTAGTGTTGCGCAA |  |  |
| 5f_9PA | CCCTGCTAGAGATTCATTGACA | 1667 | 93 |
| 6r_9PA | TCGTGACTTTACATGTTGGTGC |  |  |
| 6f_9PA | ATGCCCCTAACTCCGACAGA | 1454 | 94 |
| 7r_9PA | AACGGGTCGGGGATCTATTG |  |  |
| 7f_9PA | CGCAGCAAAAATGGTAGGAAC | 1984 | 95 |
| 8r_9PA | GAGGGTAGCTTCATGGAGCA |  |  |
| 8f_9PA | TCGCGCACAGGATGGATAAA | 819 | 96 |
| 9r_9PA | ACACCATGTAGTCCACCTGA |  |  |
| 9f_9PA | GAACCAATTTTGCCAAGGATCA | 2452 | 97A |
| 10r_9PA | TCCCGTTGCGCTAGAAAAGA |  |  |
| 10f_PA | TCGTGCCGATATCTCCAACG | 2230 | 98 |
| 11r_9PA | ACGCGCGGTACTGCTATTAT |  |  |
| 11f_9PA | GGAACAGGACCAAAAACACGA | 3106 | 99 |
| 12r_9PA | TGCTGGTTCTTATGCACGAC |  |  |
| 12f2_9PA | CCATCATTTAACTCATCACGCC | 1934 | 100A |
| 13r_9PA | ACGGTGTGTATTACTGGAGCA |  |  |
| 13f_9PA | GCCAACACCACGTAACACTC | 2172 | 101 |
| 14r_9PA | AAAGCAGAAGAGTACGGGGA |  |  |
| 14f_9PA | TAAGCCAAATCGTATATATCAC | 1114 | 102 |
| 42r_9PA | TTATGCGGCTGTTCAGCGAA |  |  |
| rRNA2_F | CGGATTCGCTCTAAGGGAGG | 7962 | rRNA2 |
| rRNA2_R | ACCTCGGTCCTTTCCACAAG |  |  |
